# Long-term entrapment and temperature-controlled-release of SF_6_ gas in metal–organic frameworks (MOFs)

**DOI:** 10.3762/bjnano.10.180

**Published:** 2019-09-10

**Authors:** Hana Bunzen, Andreas Kalytta-Mewes, Leo van Wüllen, Dirk Volkmer

**Affiliations:** 1 Chair of Solid State and Materials Chemistry, Institute of Physics, University of Augsburg, Universitätsstraße 1, D-86159 Augsburg, Germany; 2 Institute of Materials Resource Management, University of Augsburg, Universitätsstraße 1, D-86159 Augsburg, Germany; 3 Chair of Chemical Physics and Materials Science, Institute of Physics, University of Augsburg, Universitätsstraße 1, D-86159 Augsburg, Germany

**Keywords:** benzobistriazole, gas storage, kinetic trapping, metal–organic frameworks (MOFs), sulfur hexafluoride

## Abstract

In this work, a metal–organic framework (MOF), namely MFU-4, which is comprised of zinc cations and benzotriazolate ligands, was used to entrap SF_6_ gas molecules inside its pores, and thus a new scheme for long-term leakproof storage of dangerous gasses is demonstrated. The SF_6_ gas was introduced into the pores at an elevated gas pressure and temperature. Upon cooling down and release of the gas pressure, we discovered that the gas was well-trapped inside the pores and did not leak out – not even after two months of exposure to air at room temperature. The material was thoroughly analyzed before and after the loading as well as after given periods of time (1, 3, 7, 14 or 60 days) after the loading. The studies included powder X-ray diffraction measurements, thermogravimetric analysis, Fourier-transform infrared spectroscopy, scanning electron microscopy, ^19^F nuclear magnetic resonance spectroscopy and computational simulations. In addition, the possibility to release the gas guest by applying elevated temperature, vacuum and acid-induced framework decomposition was also investigated. The controlled gas release using elevated temperature has the additional benefit that the host MOF can be reused for further gas capture cycles.

## Introduction

Metal–organic frameworks (MOFs) are coordination polymers with organic ligands containing (potential) voids [1]. Their porosity and high surface area make them attractive materials for adsorption-based applications [2–5]. MOFs have been suggested as promising materials for gas storage of attractive fuel gases such as hydrogen [6–8] or methane [9–11]. In these applications the gas is adsorbed inside the pores. To enhance the guest adsorption in MOFs, several different approaches have been introduced over the last few years. These include tuning the pore properties, such as polarity, or introducing open metal sides for a better interaction between the guest and host material [5,7,11]. Recently, we reported on an alternative approach which dealt with kinetic trapping of gas molecules in MOFs [12]. This approach is based on using MOFs with ultranarrow pore apertures. Under elevated pressure and temperature, the gas molecules enter the pores, but they are not released immediately when normal conditions are re-established. This is due to the activation energy barrier for gas diffusion of the entrapped sorbate within the pores connected via ultranarrow pore apertures.

**Scheme 1:**
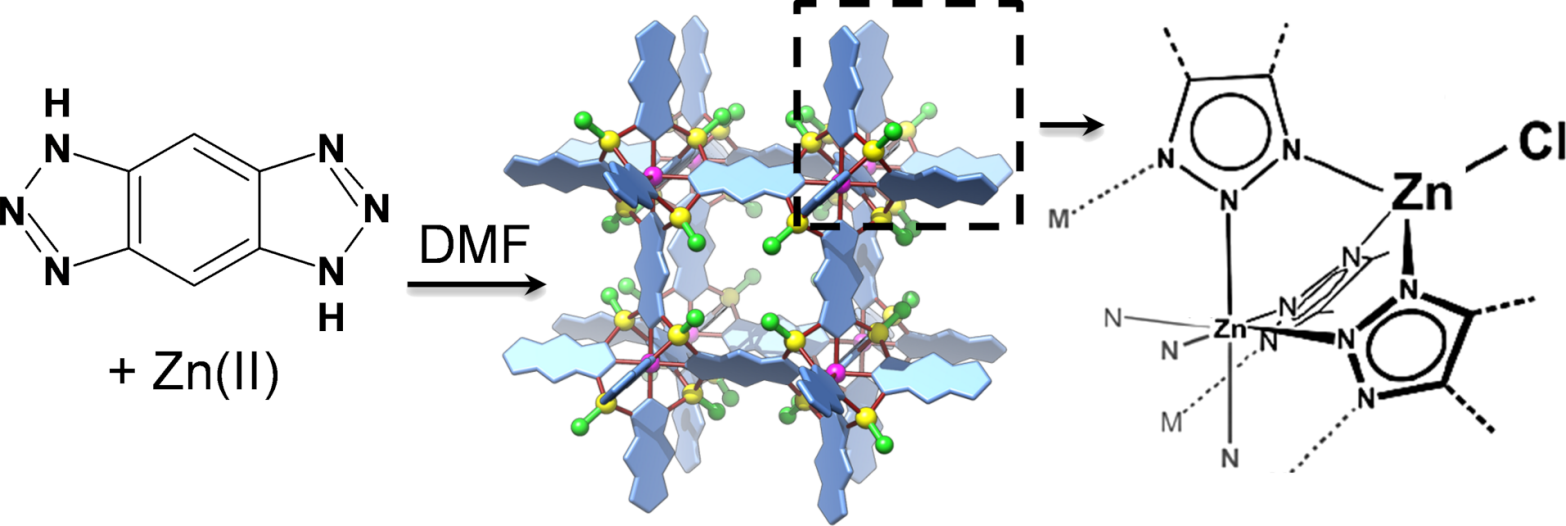
Synthesis of MFU-4.

As a proof-of-principle study, we used a MOF called MFU-4 (where MFU-4 stands for Metal−Organic Framework Ulm University-4) to trap xenon gas inside the pores [12]. MFU-4 is comprised of zinc cations and benzotriazolate ions (Scheme 1) [13]. It crystallizes in the cubic crystal system and contains two types of cavities with diameters of 3.88 and 11.94 Å, which are connected by narrow (only 2.52 Å) pore apertures. This made the MOF a promising candidate for kinetic trapping of gases. In our recent work we were able to show that it was possible to trap over 40 wt % of xenon (kinetic diameter: 3.96 Å) [14] inside the pores despite its diameter being larger than the pore aperture [12]. Upon exposure of the sample to air under normal conditions, we observed that the gas was slowly released. For instance, after three days approximately 20% of the guest gas was released and after one month more than 67% was lost. Keeping these results in mind we were curious if it was possible to permanently trap (i.e., imprison) gas inside the MOF without observing any leaking at normal conditions, thus enabling the use of MOFs as a gas storage container for dangerous gases.

In this work we selected sulfur hexafluoride (kinetic diameter: 5.50 Å) [14] as a guest, which has a much larger kinetic diameter than the previously reported entrapment of xenon gas (kinetic diameter: 3.96 Å) [14]. Additionally, its presence inside the pores can be easily followed by Fourier-transform infrared (FTIR) and ^19^F nuclear magnetic resonance (NMR) spectroscopy. SF_6_ is an inert, nonflammable and nontoxic gas, which is known to be an excellent dielectric gas for high-voltage applications [15–16]. At the same time, it is also known as one of the most severe greenhouse gases [17–18]. Therefore, there is currently much interest in finding effective materials to allow its capture that provide safe storage as well as re-use. Various porous materials have been tested for this purpose including carbons [19–20], zeolites [21–23], MOFs [24–27] and porous organic cages [28–29]. Herein we present a study applying the approach of kinetic trapping of gases in MOFs and characterize the loading of SF_6_ into MFU-4 and its release from the MOF at normal conditions over a period of two months.

## Results and Discussion

### Materials synthesis

The MOF MFU-4 (Scheme 1) was synthetized according to the procedure reported previously [12]. By carrying out the reported microwave-assisted synthesis, ≈2–10 µm cubic crystals were obtained (Figure S1 in Supporting Information File 1). Prior to guest loading, the sample was kept under vacuum at 320 °C overnight to make sure that there were no solvent molecules left in the pores, thus ensuring that the whole pore volume was available for trapping the SF_6_ guest. The bulk sample was analyzed before and after the gas loading by conventional analytical methods, including FTIR, powder X-ray diffraction (XRD) and thermogravimetric analysis (TGA).

### SF_6_-loading experiments

The SF_6_-loading was carried out at elevated pressure and temperature. To incorporate the highest amount of the guest molecules inside the pores, we tested various loading conditions. We used a pressure of 20 bar and varied the loading temperature and time. From all tested conditions (Table 1), the highest loading was achieved after 18 hours at 250 °C (sample 3). Prolonging the loading time did not lead to a higher loading. Therefore, sample 3 was used in the following studies.

**Table 1:** SF_6_-loading into MFU-4 under various conditions.

Sample	SF_6_ pressure (bar)	Temp. (°C)	Time (h)	SF_6_ loaded (wt %)^a^	Calc. no. of SF_6_ molecules per unit cell/void of MFU-4^b^

1	20	150	18	2.16	1.14 / 0.29
2	20	200	18	2.37	1.25 / 0.31
3a	20	250	18	3.09	1.65 / 0.41
3b	20	250	18	3.07	1.63 / 0.41
3c	20	250	18	3.13	1.67 / 0.42
4	20	250	48	3.04	1.62 / 0.40

^a^Mass loss in the temperature range from 150 to 390 °C determined from TGA data (see Supporting Information File 1, Figure S2). ^b^Assuming all SF_6_ molecules are located in the larger void (with four larger voids per unit cell of MFU-4).

**Figure 1:**
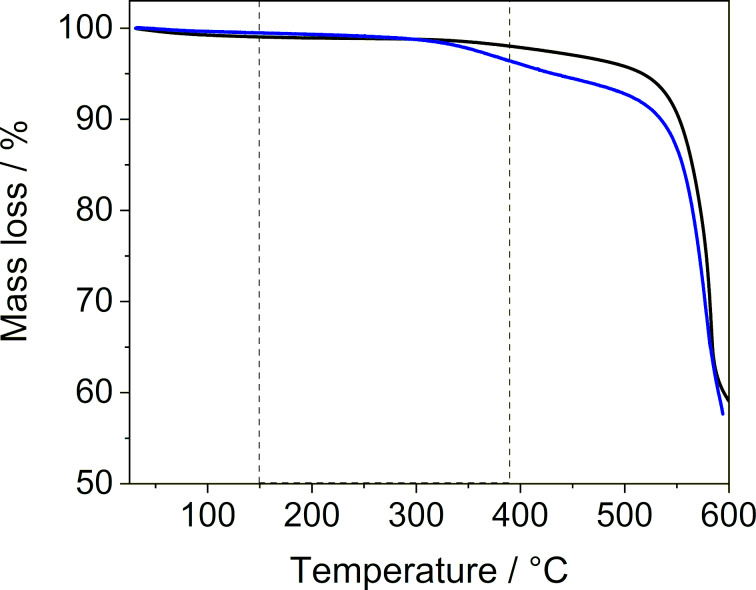
Thermogravimetric analysis of MFU-4 before (black) and after (Sample 3a, blue) the loading of SF_6_ measured under a nitrogen atmosphere at a heating rate of 10 K min^−1^.

To estimate the amount of the guest loaded into the MOF, we used TGA (Figure 1 and Figure S2 in Supporting Information File 1). The analysis revealed a gradual mass loss between 150–500 °C. When heated above 500 °C, a second mass loss was observed which corresponded to the framework decomposition. By utilizing mass spectrometry, we analyzed the gaseous products that were released during sample heating. We recorded that SF_6_ was gradually released from 150 to 390 °C (Figure S3 in Supporting Information File 1). Therefore, this temperature range was used to quantify the amount of loaded SF_6_ from the TGA data (Table 1).

The activation energy for the guest release was estimated by temperature-modulated TGA to be as high as approximately +135 kJ·mol^−1^ (Figure S4 in Supporting Information File 1). Modulated TGA (MTGA^TM^ by TA instruments) is an analytical technique used for obtaining continuous kinetic information for decomposition and volatilization reactions. The method makes use of an oscillation temperature program to obtain kinetic parameters during a mass loss [30–31]. Until now it has been mainly used to study organic polymers (e.g., poly(ethylene) and poly(styrene)) and simple inorganic compounds (e.g., calcium carbonate and calcium oxalate) [31]. Here we used the method to estimate the activation energy of a guest released from a porous material. The determined value of the activation energy was further compared to the results obtained from computational simulations (see the dedicated section later in the text).

**Figure 2:**
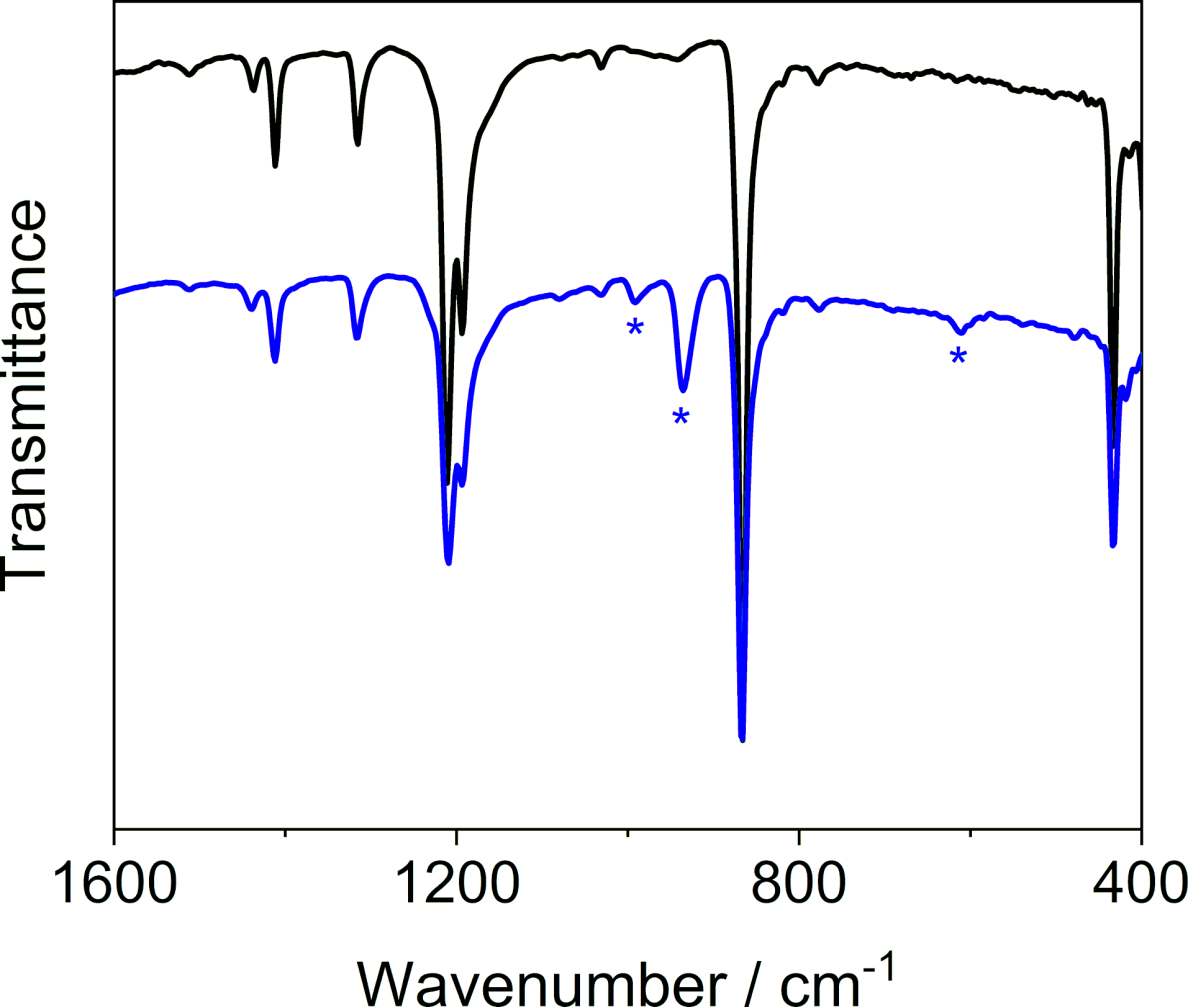
FTIR spectra of MFU-4 before (black) and after (Sample 3a, blue) the loading of SF_6_. Bands attributed to SF_6_ are marked with an asterisk.

To qualify the guest, we used FTIR spectroscopy (Figure 2 and Figure S5 in Supporting Information File 1). The FTIR spectrum revealed, beside the bands originating from MFU-4, three additional bands at 610.8, 935.4 and 991.6 cm^−1^. These bands can be assigned to the *v*_4_ (liquid: 610.8 cm^−1^; gas: 614.5 cm^−1^), *v*_3_ (liquid: 914.9 cm^−1^; gas: 948.0 cm^−1^) and *v*_2_ + *v*_6_ (liquid: ≈990 cm^−1^; gas: 984.2 cm^−1^) vibrational modes in SF_6_ [32].

**Figure 3:**
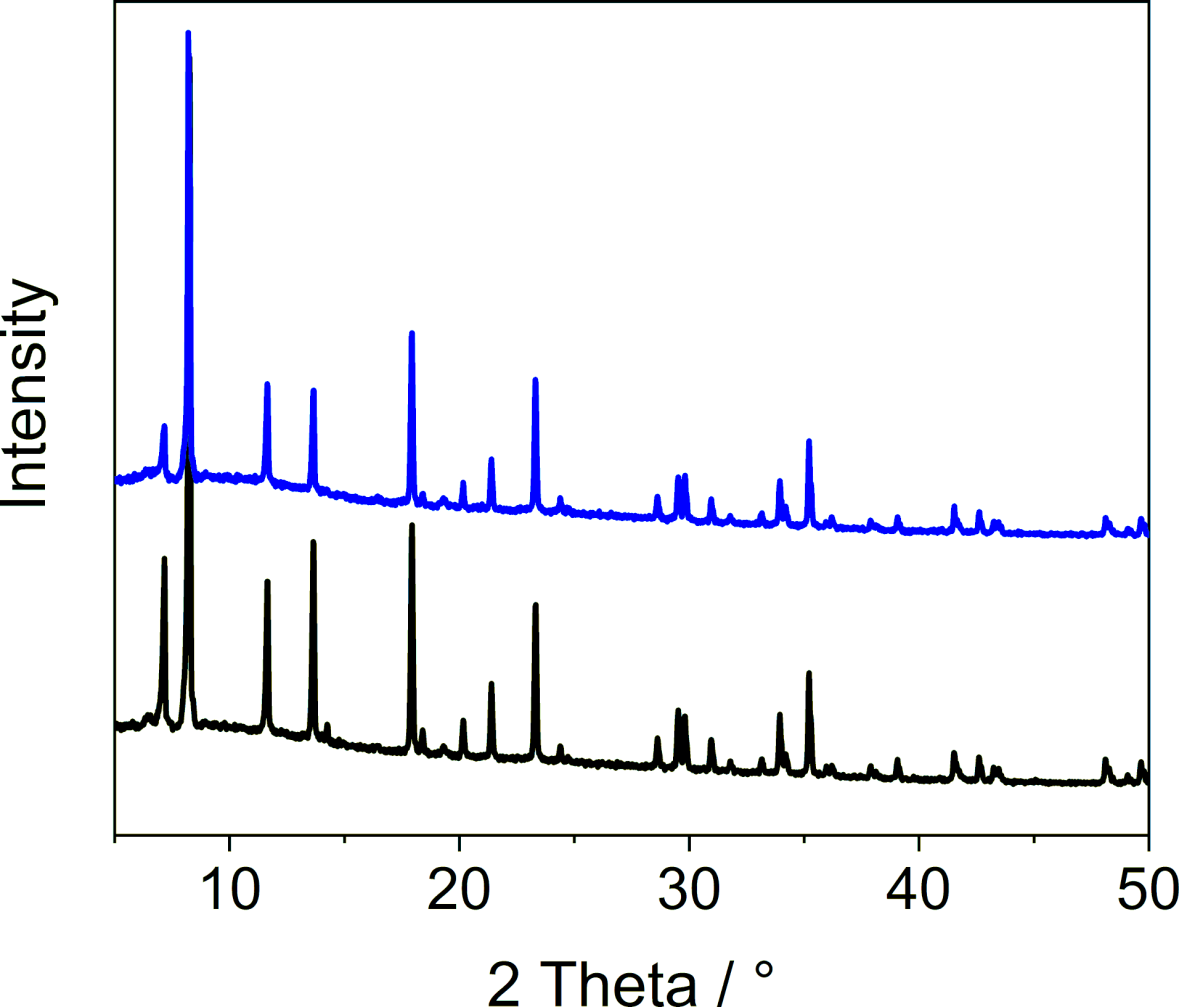
Powder X-ray diffraction analysis of MFU-4 before (black) and after (Sample 3a, blue) the loading of SF_6_.

To check that the sample crystallinity was preserved, we measured powder XRD patterns before and after the loading (Figure 3). The recorded powder XRD patterns did not reveal any changes in the diffraction peak positions, but there were differences in the peak intensities. Similar variations in the peak intensities of MOF samples have been previously described and assigned to the presence of solvent molecules inside the pores [33]. Therefore, here the changes in intensities can be seen as a sign of SF_6_ molecules being successfully included into the pores. Last but not least, scanning electron microscopy (SEM) images of the sample (taken before and after the guest loading) did not reveal any detectable changes in the crystal surface and morphology, confirming that the MOF crystals remained intact during the loading (Supporting Information File 1, Figure S1).

**Figure 4:**
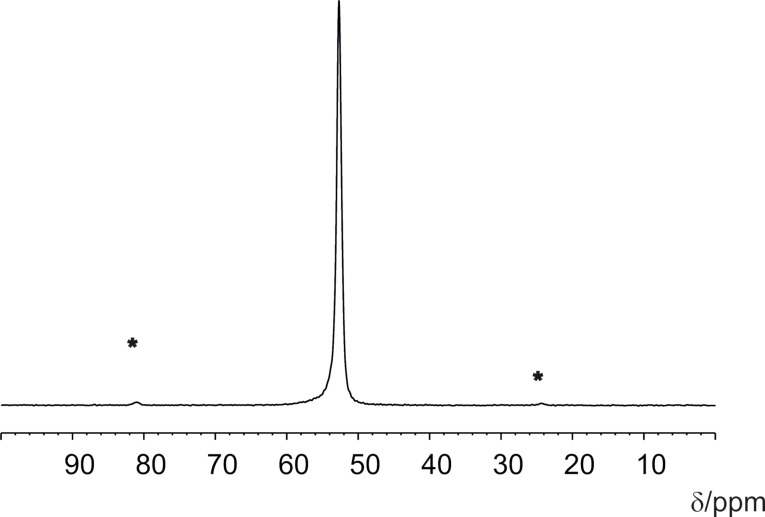
^19^F MAS NMR spectrum of MFU-4 (Sample 3b) loaded with SF_6_ recorded at room temperature. The spectrum was referenced against CFCl_3_.

The presence of SF_6_ could be further confirmed by NMR measurements. The ^19^F MAS NMR spectrum, obtained at a MAS frequency of 8 kHz, revealed one singlet signal at 52.7 ppm, which can be assigned to the ^19^F nuclei of the SF_6_ guest (Figure 4). The line position is approximately 5 ppm upfield from the resonance of gaseous SF_6_ (57.42 ppm) [34]. Additionally, small signals (marked by asterisks in Figure 4) to the left and right side of the main singlet represent spinning sidebands, which illustrates that the SF_6_ molecule is not completely freely rotating and that there is an interaction with the MOF host lattice.

### Computational simulations

**Figure 5:**
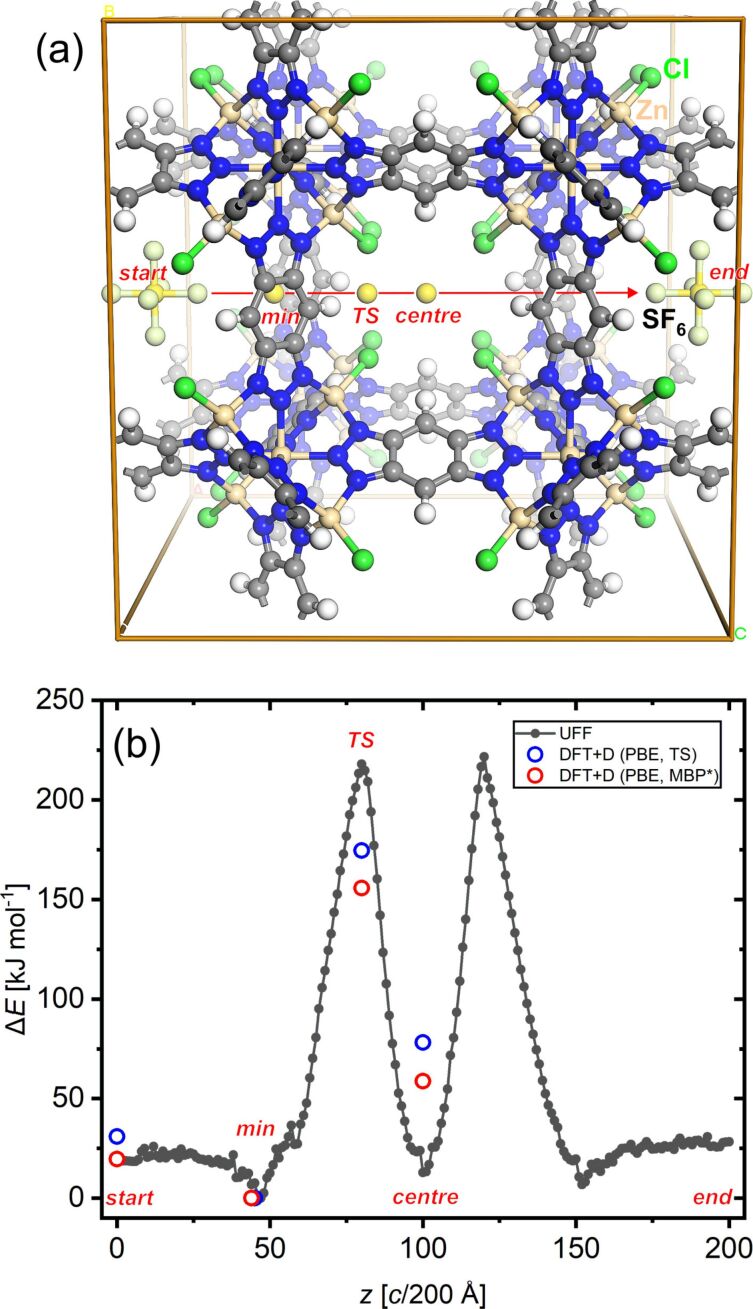
(a) MFU-4 unit cell showing the linear transition scan path for SF_6_ crossing a single small pore. (b) Black curve and filled circles: differences of UFF total energy values of 200 configurations of SF_6_@MFU-4 scanned along the linear path displayed in (a). Open circles: differences of DFT total energy values for selected configurations and different dispersion correction methods (for explanations see text).

Theoretical calculations were performed in order to determine the activation energy parameters from atomistic simulation data. Briefly, the approach previously described for scanning the minimum energy path of xenon atoms crossing the small pore in MFU-4 [13] was adapted and further refined in order to account for the multiatomic nature of the diffusant, i.e., SF_6_. The linear transition path of SF_6_ passing through a single unit cell of MFU-4 is shown in Figure 5a, which shows the start and end position of a SF_6_ molecule, serving as a probe for scanning the potential energy of the system along the displayed path. To obtain a rough estimate for the energy changes along the path, molecular mechanics calculations were performed, employing the universal force field (UFF) [35] parameters as included in the GULP code [36]. In order to prevent an origin shift of the framework during transition path sampling, the fractional atom coordinates of four framework hydrogen atoms were fixed. In addition, the geometry of the linear F–S–F group (as part of SF_6_) parallel to the transition path was restrained to the UFF equilibrium bond distance values. During transition path sampling all unit cell parameters were held constant. All other atom positions were allowed to relax freely during the simulation. An example input file is provided as part of the Supporting Information File 1.

The change of the total energy of all 200 configurations sampled during the linear transition of SF_6_ through the unit cell of MFU-4 is shown as the black curve in Figure 5b. The potential energy curve is symmetric, showing two pronounced energy maxima at the transition path scan coordinates *c*/80 (marked by “TS”) and *c*/120, respectively. At these coordinates the SF_6_ molecule experiences the highest repulsive interaction energy, thus leading to a strong geometric distortion of the small pores of MFU-4. The energy minima are located at *c*/47 (marked by “min”) and *c*/152; According to force field calculations, another (local) energy minimum is found in the middle of the pore (*c*/100, “centre”), which is surprising in light of the close-packed arrangement of coordinated chloride ligands in the framework structure. According to the potential energy curve, an activation energy of approximately +218 kJ·mol^−1^ can be estimated, which is significantly higher than the experimental value of +135 kJ·mol^−1^, as gleaned from the temperature-modulated thermogravimetric analysis.

In order to obtain more realistic energy values, constrained geometry plane wave DFT+D calculations were performed for four different configurations (“min”, “TS”, “centre” and “start/end”) as marked in Figure 5a. For this, the starting configurations were extracted from the previous force field scan trajectory and all atomic positions were allowed to relax during subsequent optimization steps, except for the position of the sulfur atom of SF_6_, which was fixed at the corresponding *c*/N coordinate of the transition path. The PW-DFT+D calculations were performed with the CASTEP code [37] employing the PBE functional [38] and on-the-fly generated ultrasoft pseudopotentials (energy cutoff: 570 eV). Two different correction methods were included in all DFT calculations in order to account for dispersion interactions. The total energy values of these are shown in Figure 5b as blue (TS dispersion correction [39]) and red circles (MBD* dispersion correction [40]). The latter dispersion correction scheme leads to an approximate DFT-calculated activation energy of +156 kJ·mol^−1^, which is in good agreement with the experimentally determined value, taking into account the fact that all calculated values formally correspond to a temperature of zero Kelvin. In the future, metadynamic MD simulation studies might be performed, which should take into account both the effects of different gas loading conditions as well as temperature-dependent lattice vibrations and distortions. For this purpose, a well-parametrized force field for the MFU-4 host lattice and the diffusant will have to be developed.

### SF_6_-release experiments

**Figure 6:**
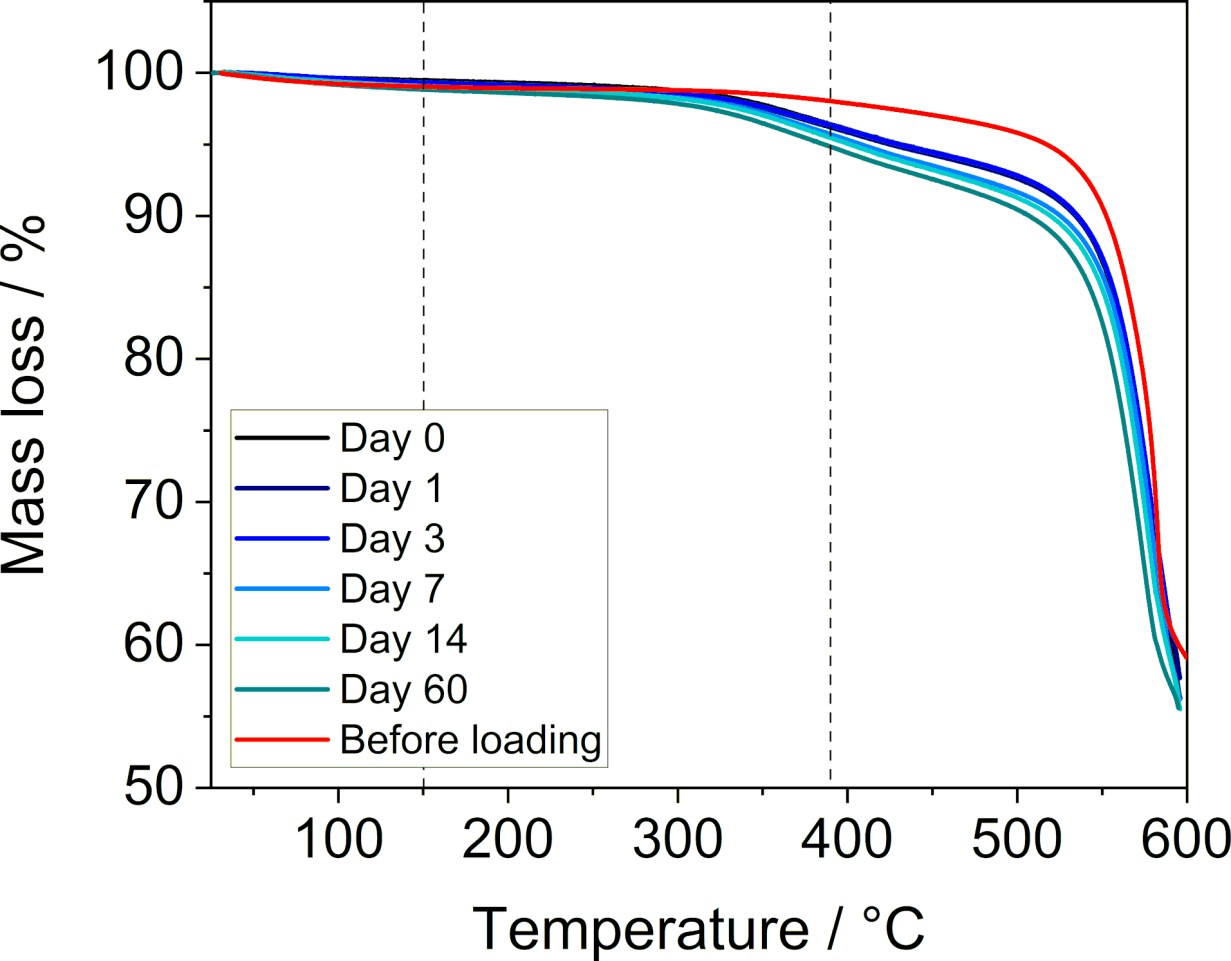
Thermogravimetric analysis of MFU-4 loaded with SF_6_ (Sample 3a) after 0, 1, 3, 7, 14, and 60 days measured under a nitrogen atmosphere at a heating rate of 10 K min^−1^.

**Figure 7:**
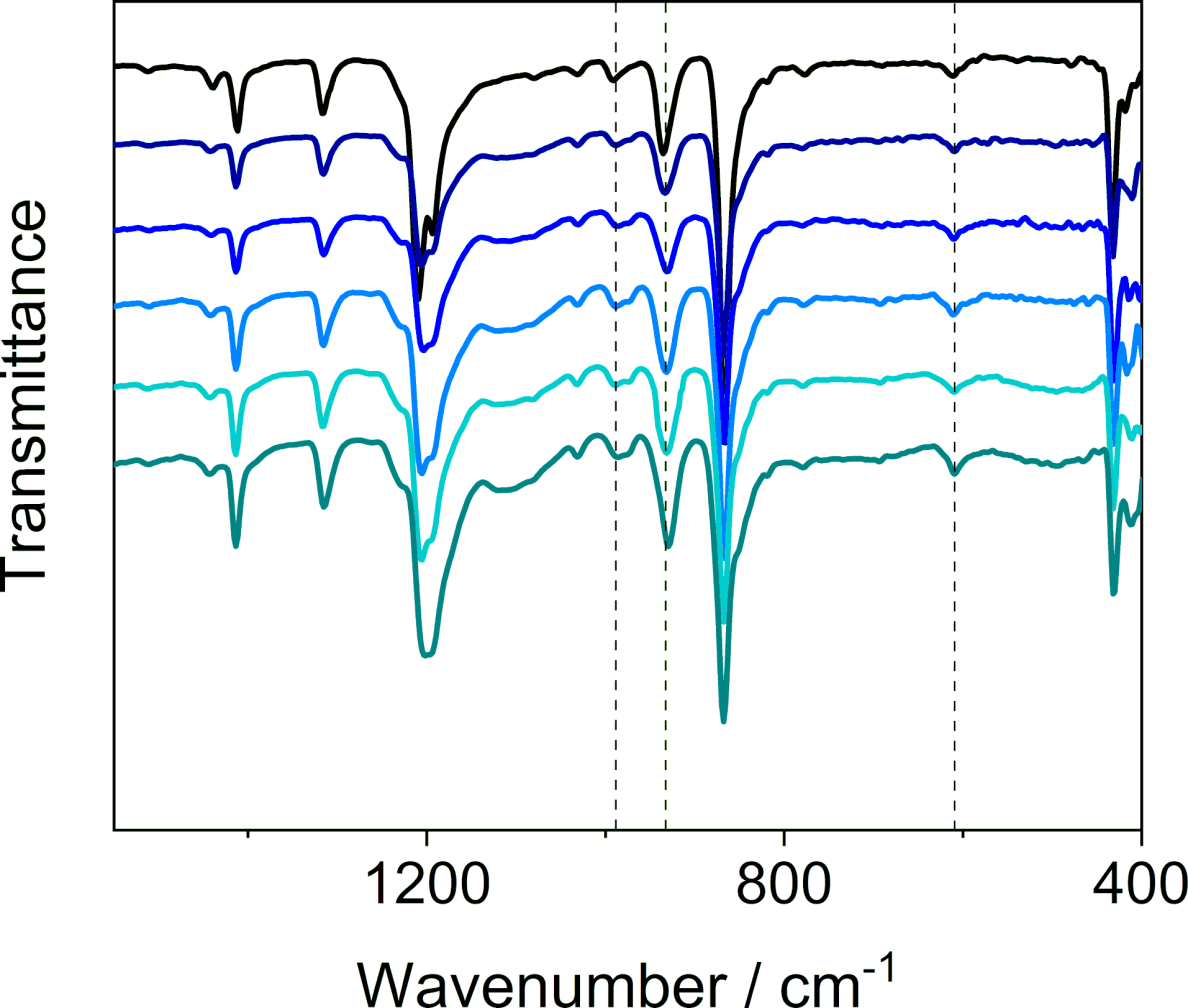
FTIR spectra of MFU-4 loaded with SF_6_ (Sample 3a) after 0, 1, 3, 7, 14, and 60 days. Bands attributed to SF_6_ are marked with dotted vertical lines.

The SF_6_-loaded MFU-4 sample was stored in air at room temperature. After a certain period (0, 1, 3, 7, 14 and 60 days) the sample was analyzed by TGA (to quantify the amount of the guest in MFU-4, Figure 6), powder XRD measurements (Figure S6 in Supporting Information File 1) and FTIR spectroscopy (Figure 7 and Figure S7 in Supporting Information File 1). Based on the results of these measurements, it can be concluded that there was no leaking of the guest. Both TGA (Figure 6 and Table 2) and FTIR spectroscopy (Figure 7, and Figure S8 and Table S1 in Supporting Information File 1) showed that SF_6_ remained inside the pores over the entire investigated period. The analyses also revealed that upon exposure of the sample to air, a small amount of water was adsorbed onto the surface of the MFU-4 crystals.

**Table 2:** SF_6_ release from MFU-4 (Sample 3a) stored in air at room temperature for 0–60 days as evaluated by thermogravimetric analysis (Figure 6).

Time (days)	H_2_O adsorbed (wt %)^a^ (25–150 °C)	SF_6_ loaded (wt %)^a^ (150–390 °C)	Calc. no. of SF_6_ molecules per unit cell/void of MFU-4^b^

0	0.53	3.09	1.65 / 0.41
1	0.67	3.08	1.64 / 0.41
3	0.75	3.03	1.61 / 0.40
7	0.91	3.10	1.65 / 0.41
14	0.95	3.00	1.60 / 0.40
60	1.22	3.29	1.76 / 0.44

^a^Determined by TGA (Figure 6). ^b^Assuming all SF_6_ molecules are located in the larger void (with four larger voids per unit cell of MFU-4).

As indicated by the results of the thermogravimetric analysis (Figure 1), if needed, the guest gas can be released in a controlled manner by heating the sample without decomposing the framework. This was proven by a variable temperature powder XRD measurement (Figure S9 in Supporting Information File 1), which confirmed that MFU-4 was stable up to 500 °C. This temperature is higher than that needed for the SF_6_ release, enabling the host material to be reused for further guest storage. To show that SF_6_ can be released without its decomposition, a study of temperature-induced gas release followed by mass spectrometry was carried out. In this measurement only signals corresponding to the SF_6_ fragments (such as SF_5_^·^ and SF_4_^·^) [41] and no signals of its thermal decomposition products [42] could be detected, confirming that the gas release from the host material was possible (for details see Figure S3 in Supporting Information File 1).

In another experiment, we examined if it was possible to release the gas guest at room temperature by applying vacuum. We kept a sample of MFU-4 loaded with SF_6_ (Sample 3c) under vacuum at 25 °C for a certain period of time and then analyzed it by FTIR and TGA (Figure S10 and S11 in Supporting Information File 1). Even after 24 h of applying high vacuum (*p* = 2.8 × 10^−7^ mbar), we could still detect a substantial amount of SF_6_ which corresponded to approximately 90% of the originally loaded amount (determined by TGA; see Figure S11 in Supporting Information File 1). Furthermore, we studied the possibility to release SF_6_ from the pores of MFU-4 by decomposing the framework. To do so, we treated a sample of the MOF loaded with SF_6_ with an acid, namely concentrated sulfuric acid, and analyzed the gas phase by mass spectrometry (Figure S12 in Supporting Information File 1). As expected, upon adding the acid to the reaction vessel, the framework decomposed and an immediate evolution of gas bubbles was observed. The SF_6_ release could be followed by mass spectrometry; however, a precise quantification was not possible with the experimental set-up used. SF_6_ is a heavy gas (MW: 146.06 g·mol^−1^), and thus some of the gas remained in the bottom of the reaction vessel and possibly also dispersed as bubbles in the viscous reaction solution.

## Conclusion

In this study we were able to show that it was possible to trap SF_6_ gas in a MOF and that the gas guest remained inside the MOF upon exposing the sample at room temperature to air. Even after two months we did not observe any measurable leaking of the guest gas from the host. Furthermore, even under high-vacuum conditions, most (90%) of the gas remained entrapped in the pores. This finding could lead to a new gas storage method for dangerous gasses. We also showed that it was possible to release the gas guest in a controlled manner by applying elevated temperature or by decomposing the material by acid digestion. For gas release by elevated temperature, the host MOF can be recycled for further gas-capture cycles. In the future we plan to study the influence of the material crystal size on the loading capacity and focus on engineering structural properties of MFU-4 in order to prepare its analogues with different pore aperture sizes. We believe that such materials could find potential application in gas storage and separation.

## Experimental

Benzobistriazole was synthetized according to the previously reported procedure [43]. Anhydrous ZnCl_2_ and DMF were of analytical grade and used as-received from commercial suppliers. SF_6_ (≥99.995+%) was also used as-received from a commercial supplier (Linde). FTIR spectra were recorded in the range of 400–4000 cm^−1^ on a Bruker Equinox 55 FTIR spectrometer equipped with an ATR unit. Thermogravimetric analysis (TGA) was measured on a TA Instruments Q500 device over a temperature range of 25–600 °C under a nitrogen atmosphere at a heating rate of 10 K min^−1^. Temperature-modulated TGA was measured on a TA Instruments Q500 device in the temperature range of 25–550 °C under helium atmosphere at a heating rate of 1.5 K min^−1^, amplitude of ±5 °C and period of 200 s. The temperature-induced gas release was followed by a BelCat-B catalyst analyzer (Bel Japan, Inc.) coupled with a mass spectrometer (OmniStar GSD 320, Pfeiffer Vacuum). The sample was placed between two plugs of quartz wool in a quartz glass reactor and heated up to 500 °C (10 K·min^−1^) under a flow of helium (30 mL·min^−1^). The composition of the exhaust gas was analyzed by a mass spectrometer. The acid-induced gas release was carried out in a round-bottom flask purged with a flow of argon (100 mL·min^−1^) and connected to a BelCat-B catalyst analyzer (Bel Japan, Inc.) attached to a mass spectrometer (OmniStar GSD 320, Pfeiffer Vacuum). To a solid sample (20 mg), 1 mL of concentrated sulfuric acid (95–98 wt %) was added and the gas phase was analyzed by the mass spectrometer. Powder XRD data were collected in the 5–50° 2θ range using a Seifert XRD 3003 TT powder diffractometer with a Meteor1D detector operating at room temperature using Cu Kα_1_ radiation (λ = 1.54187 Å). Variable-temperature powder XRD data were measured in the 5–50° 2θ range under nitrogen atmosphere with an Empyrean (PANalytical) diffractometer equipped with a Bragg–Brentano HD mirror and a PICcel3D 2×2 detector. The temperature program was carried out at 50 °C steps up 600 °C at a heating rate of 0.5 °C s^−1^ and held for 10 min between the measurements. SEM micrographs were recorded on a Zeiss Gemini 2 (Crossbeam 550) instrument operating at 30 kV. The ^19^F MAS NMR spectrum was recorded at a frequency of 282.5 MHz employing a Bruker Avance III spectrometer at a field of 7 T equipped with a 4 mm Bruker MAS probe. The MAS frequency was set to 8 kHz, and a repetition rate of 2 s (*T*_1_ = 0.37 s at room temperature) was used to collect the signal. The spectrum was referenced against CFCl_3_.

### MFU-4 synthesis

MFU-4 was prepared by a microwave-assisted synthesis following the previously reported procedure [13]. Briefly, a mixture of benzobistriazole (100 mg, 0.624 mmol) and anhydrous ZnCl_2_ (340 mg, 2.495 mmol) in DMF (5 mL) was placed in a pyrex tube (30 mL). The reaction mixture was heated in a microwave synthesizer (CEM, Discover S) to 155 °C at 300 W and kept under these conditions for 10 min and then cooled to room temperature. The formed precipitate was isolated by centrifugation, washed well with DMF (3 × 5 mL) and dried in air at ambient conditions to give an off-white crystalline material (166 mg). Prior to the SF_6_-loading experiments, the material was kept under vacuum at 320 °C overnight to remove any solvent molecules from the voids.

### SF_6_-loading experiments

Analogous as described in [12], in each experiment, 50−100 mg of MFU-4 was placed in a steel vessel constructed from metal tubing attached to a manometer. The vessel was filled with SF_6_ gas and kept at the desired pressure and temperature for a desired period of time. Upon cooling, the gas pressure was released, and the sample was immediately analyzed with TGA, FTIR and XRPD methods.

### SF_6_-release experiments

In a similar manner as described in [12], the SF_6_-loaded sample was stored in a container opened to air, and after a certain period of time (0, 1, 3, 7, 14 or 60 days) a small amount (≈10 mg) was taken and analyzed by TGA, FTIR and XRPD methods.

### Computational simulations

To obtain a rough estimate of the energy changes along the linear transition path of SF_6_ passing through a single unit cell of MFU-4 (Figure 5a), molecular mechanics calculations were performed, employing the universal force field (UFF) [35] parameters as included in the GULP code [36]. Force field atom types were assigned automatically within Material Studio’s Visualizer GUI [37]. The cell parameters for the cubic unit cell of MFU-4 were taken from the published single crystal structural data, with *a* = 21.697 Å [13], which was kept at the experimental value in all subsequent calculations. The electrostatic-potential-derived partial (ESP) charges for the lattice atoms of MFU-4 and for SF_6_ were obtained from discrete cluster DFT calculations, as described previously [44]. ESP values for the symmetry unique atoms of MFU-4 and SF_6_ are displayed in the Supporting Information File 1 in Figure S13.

Prior to the potential energy scan, all atomic positions of the MFU-4 unit cell were fully relaxed at tight convergence settings. Next, a single SF_6_ molecule was added to the unit cell, centered at a fractional atomic position 0.5*a*, 0.5*b*, 0.0*c*. The transition path of SF_6_ was set to the length of one unit cell and a complete translation of SF_6_ was performed in 200 steps, ending at fractional atomic positions 0.5*a*, 0.5*b*, 1.0*c.* The “translation” directive included in GULP was employed for automatizing the SF_6_ linear transition task in a single run (setting: translate 0.0 0.0 1.0 200 noise 0.05), during which a geometry-constrained linear F–S–F fragment placed along the transition path (as part of SF_6_) was moved in steps of *c*/200 Å in the <001> direction. The lattice constants and some hydrogen atoms were fixed during the run, the latter constraints being required to avoid translation of the whole framework when approaching the transition state of the scan path. An example input file is provided as part of Supporting Information File 1. PW-DFT+D calculations were performed with the CASTEP code [37], employing the PBE functional [38] and on-the-fly generated ultrasoft pseudopotentials (energy cut-off: 570 eV). Geometry optimizations were performed in different symmetry-constrained unit cells of MFU-4 (tetragonal space group *P*4mm (no. 99) for those cells corresponding to “min”, “TS”, and “centre”; cubic space group *P*m−3m for the “start/end” configuration). Again, the experimental lattice parameter *a* = 21.697 Å was retained during all calculations. The two different correction methods were included in all DFT calculations in order to account for dispersion interactions.

## Supporting Information

File 1 Additional results of SF_6_-loading and SF_6_-release experiments (TGA, FTIR and powder XRD measurements), and an example of an input file for the computation simulations.
