# Supplementary material for: Long-term entrapment and temperature-controlled-release of SF6 gas in metal–organic frameworks (MOFs)
Source: Beilstein J Nanotechnol. 2019 Sep 10;10:1851–9. doi: 10.3762/bjnano.10.180 (PMC6753670; doi:10.3762/bjnano.10.180)
Supplement: File 1 — Additional results of SF6-loading and SF6-release experiments (TGA, FTIR and powder XRD measurements), and an example of an input file for the computation simulations. [file Beilstein_J_Nanotechnol-10-1851-s001.pdf]

BEILSTEIN JOURNAL OF NANOTECHNOLOGY

Supporting Information

for

Long-term entrapment and temperature-controlled-release of SF_6 gas in metal–organic frameworks (MOFs)

Hana Bunzen, Andreas Kalytta-Mewes, Leo van Wüllen and Dirk Volkmer

Beilstein J. Nanotechnol. **2019,** *10,* 1851–1859. doi:10.3762/bjnano.10.180

Additional results of SF_6-loading and SF_6-release experiments (TGA, FTIR and powder XRD measurements), and an example of an input file for the computation simulations

License and Terms: This is a supporting information file under the terms of the Creative Commons Attribution License (http://creativecommons.org/licenses/by/4.0). Please note that the reuse, redistribution and reproduction in particular requires that the authors and source are credited.
The license is subject to the *Beilstein Journal of Nanotechnology* terms and conditions: (https://www.beilstein-journals.org/bjnano)

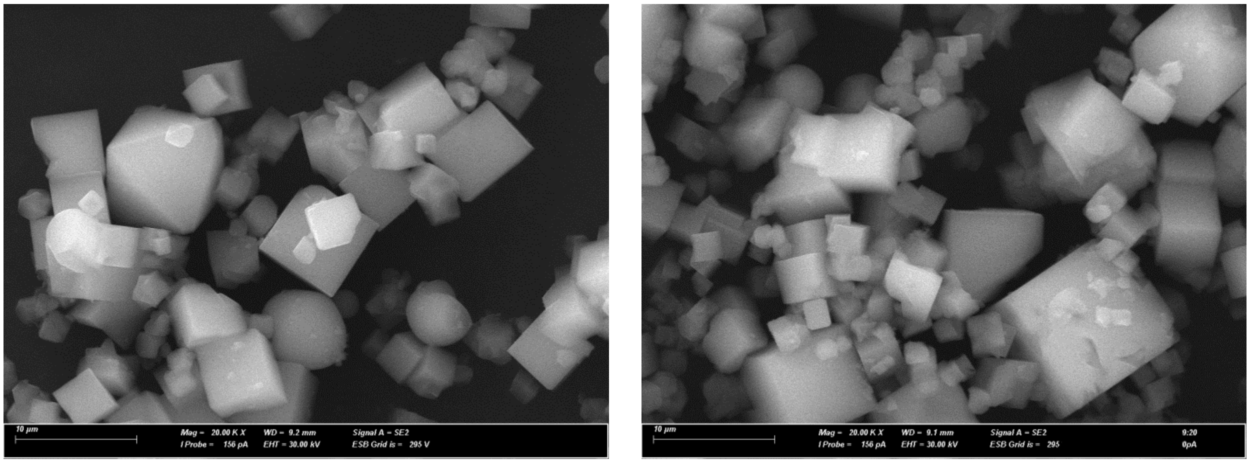

Figure S1: SEM micrographs of MFU-4 crystals before (left) and after (right, Sample 3a) the loading of SF_6; scale bar: 10 µm.

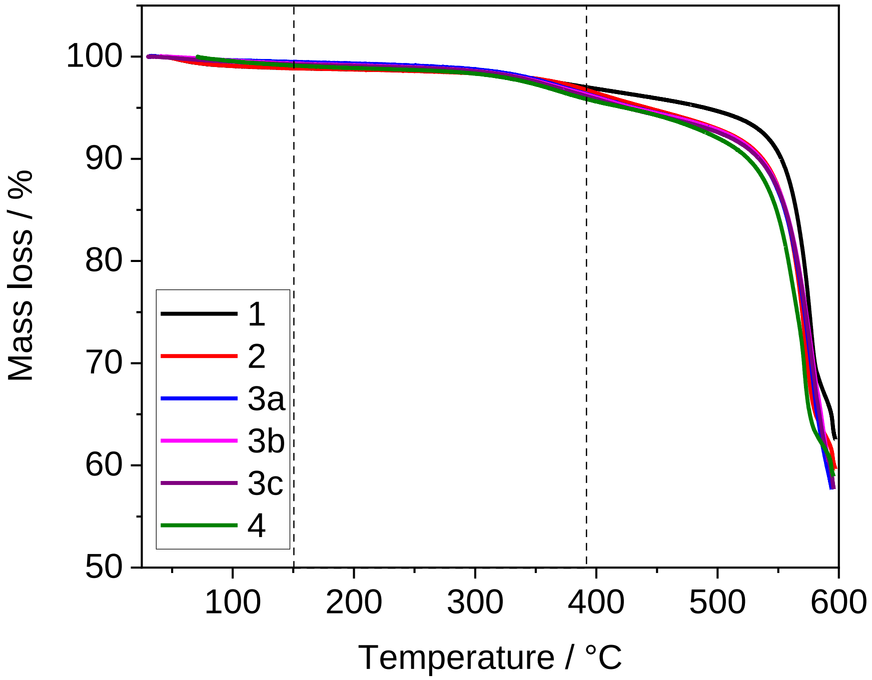

Figure S2: TG analysis of MFU-4 loaded with SF_6 (Samples 1–4 shown in Table 1) measured under a nitrogen atmosphere at a heating rate of 10 K min^{-1}.

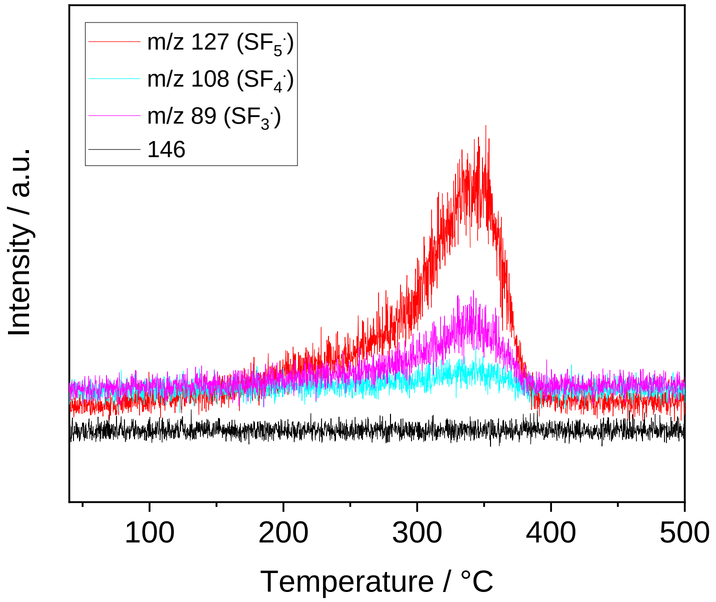

Figure S3: Temperature induced gas release study from MFU-4 loaded with SF_6 (Sample 3b) carried out under a helium atmosphere followed by mass spectrometry (EI).
Signals detected (*m/z*): 127 ($SF_5^{\cdot}$), 108 ($SF_4^{\cdot}$) and 89 ($SF_3^{\cdot}$). Signals not detected (m/z), for instance: 146 ($SF_6^{\cdot}$); 124 ($SOF_4^{\cdot}$), 102 ($SO_2F_2^{\cdot}$) and 86 ($SOF_2^{\cdot}$); 34 ($H_2S^{\cdot}$) and 20 (HF); 119 ($C_2F_6^{\cdot}$) and 69 ($CF_4^{\cdot}$).

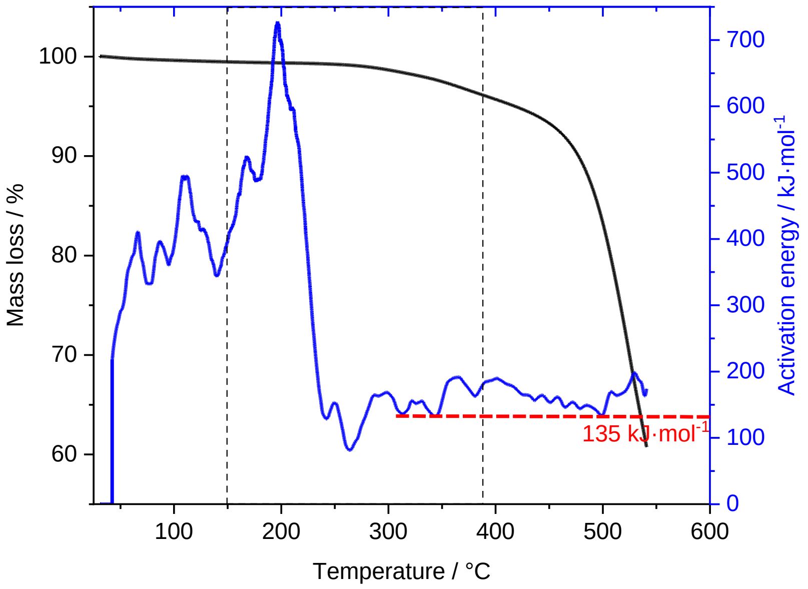

Figure S4: Temperature-modulated TG analysis of MFU-4 loaded with SF_6 (Samples 3) measured under a helium atmosphere at a heating rate of 1.5 K min^{-1}, amplitude of ± 5 °C and period of 200 s.

Modulated thermogravimetric analysis (MTGA^{TM} by TA Instruments) is an analytical technique used for obtaining continuous kinetic information for decomposition and volatilization reactions. The method makes use of an oscillation temperature program to obtain kinetic parameters during a mass loss [1].

The continuous activation energy curve resulting from the linear heating rate is shown in Figure S3. When there is no mass loss, the activation energy is set to zero. Once the onset of mass loss is observed, calculation of kinetic parameters is initiated. Because it takes several cycles to produce reliable data, the first few data points are unrealistically high.

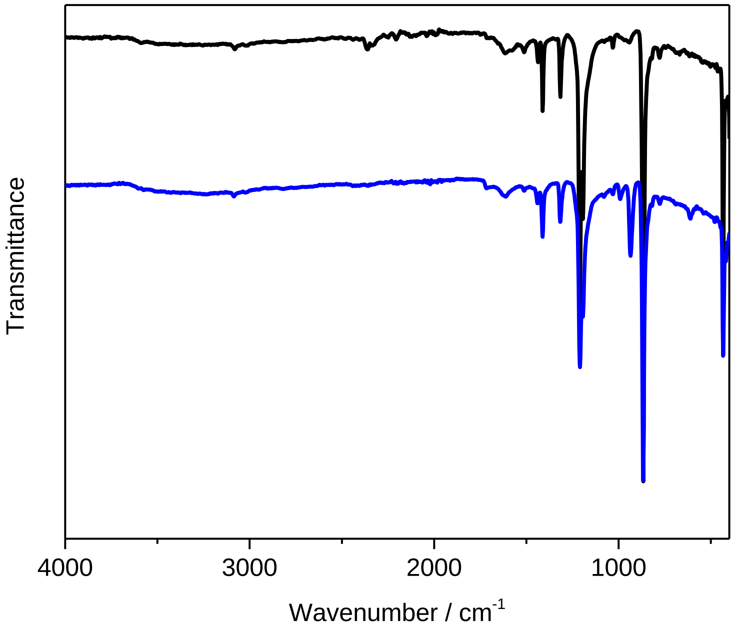

Figure S5: FTIR spectra of MFU-4 before (black) and after (Sample 3a, blue) the loading of SF_6.

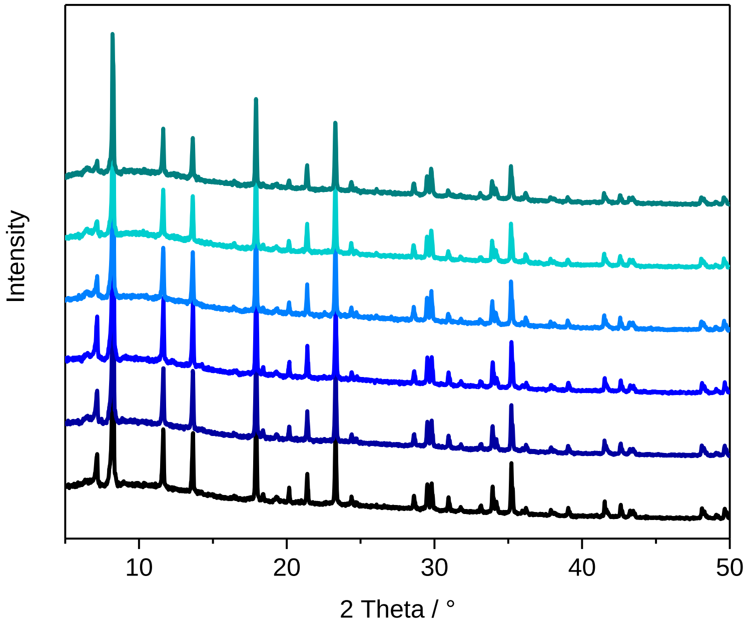

Figure S6: XRPD analysis of MFU-4 before (black) and after (Sample 3a, blue) the loading of SF_6.

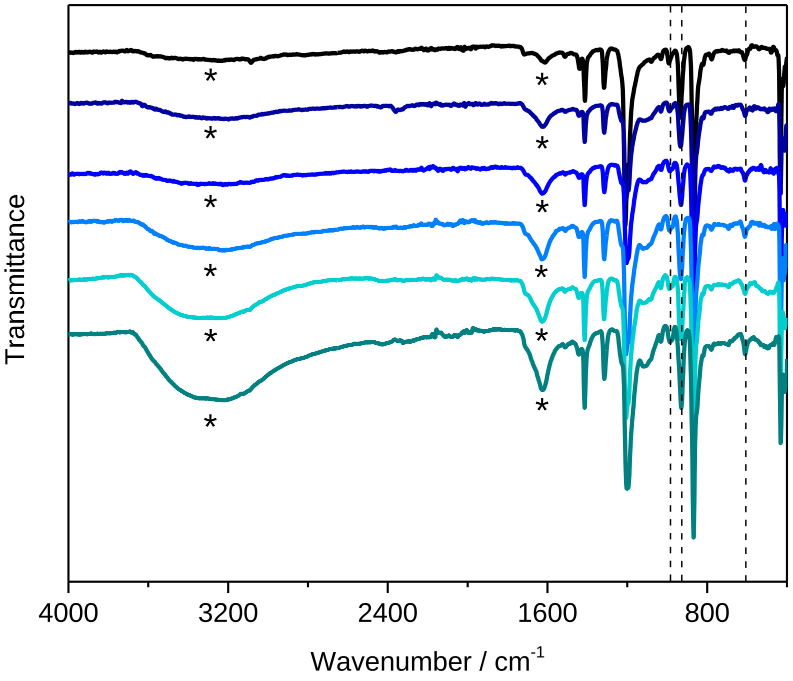

Figure S7: FTIR spectra of MFU-4 loaded with SF_6 (Sample 3a) after 0, 1, 3, 7, 14, and 60 days. Bands attributed to SF_6 are marked with lines; bands attributed to H_2O are marked with an asterisk.

Table S1: Release of SF_6 from MFU-4 followed by FTIR spectroscopy (Figure S7).

Time	MFU-4 band (ν_{max} = 1316 cm^{-1}) Area I (1334 – 1285 cm^{-1})	SF_6 band (ν_{max} = 935 cm^{-1}) Area II (960 – 899 cm^{-1})	Area I / Area II
Day 0	0.43010	0.98704	0.436
Day 1	0.29790	0.68587	0.434
Day 3	0.24544	0.53837	0.456
Day 7	0.44067	1.01079	0.436
Day 14	0.90495	2.03386	0.445
Day 60	1.04635	2.30517	0.454

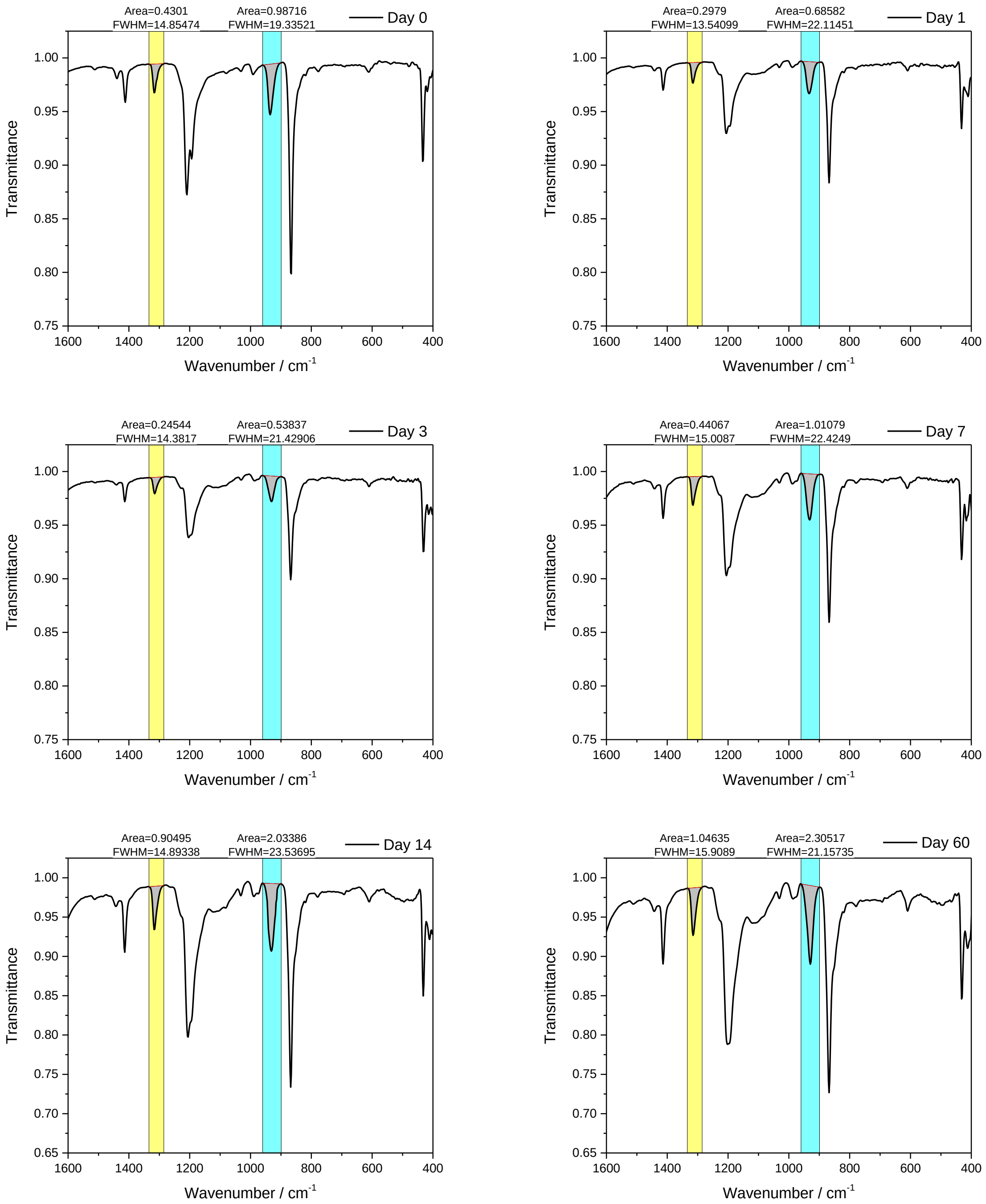

Figure S8: FTIR spectra of MFU-4 loaded with SF_6 (Sample 3a) after 0, 1, 3, 7, 14, and 60 days showing integration of the bands at v_{max} = 1316 cm^{-1} (MFU-4, yellow) and v_{max} = 935 cm^{-1} (SF_6, blue).

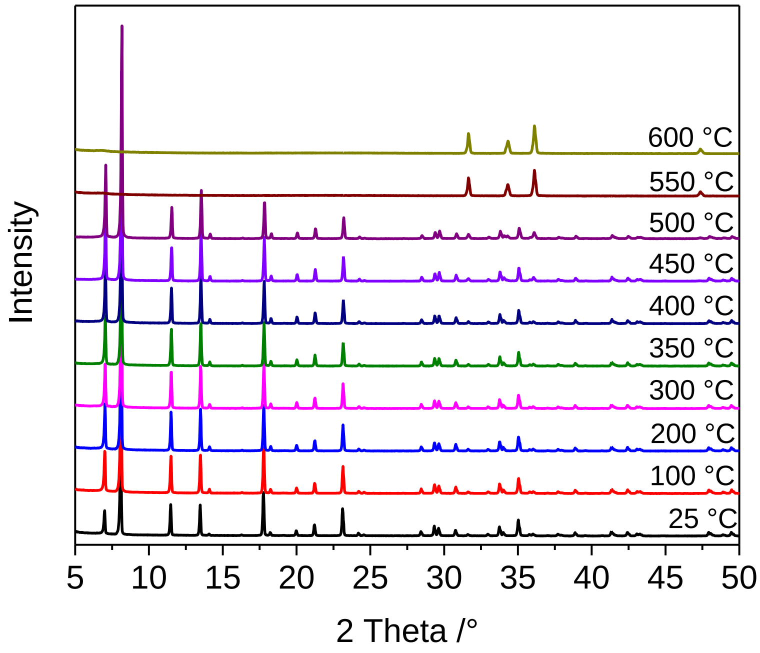

Figure S9: VT XRPD analysis of MFU-4 loaded with SF_6 (Sample 3b) carried out under a nitrogen atmosphere.

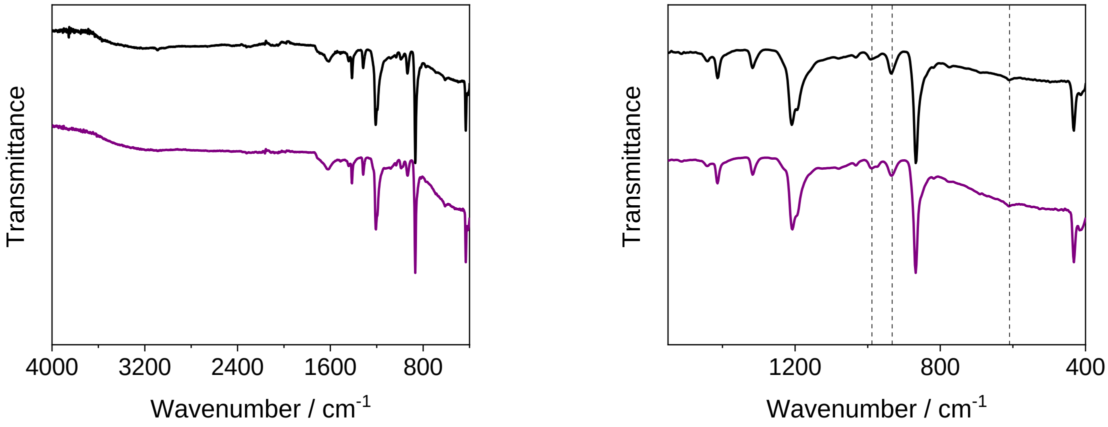

Figure S10: FTIR spectra of MFU-4 loaded with SF_6 (Sample 3c) before (black line) and after (purple line) keeping the sample under vacuum ($p = 2.8 \times 10^{-7}$ mbar) at room temperature for 24 h; bands attributed to SF_6 are marked with lines.

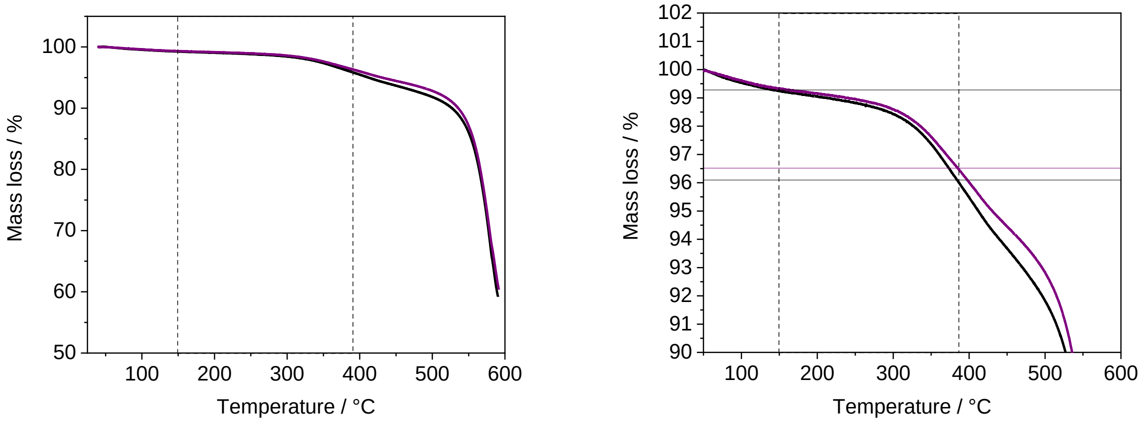

Figure S11: TG analysis carried out under a nitrogen atmosphere at a heating rate of 10 K min^{-1} of MFU-4 loaded with SF_6 (Sample 3c) before (black line) and after (purple line) keeping the sample under vacuum ($p = 2.8 \times 10^{-7}$ mbar) at room temperature for 24 h; mass loss (150–390 °C): black line – 3.13 wt %, purple line – 2.81 wt %.

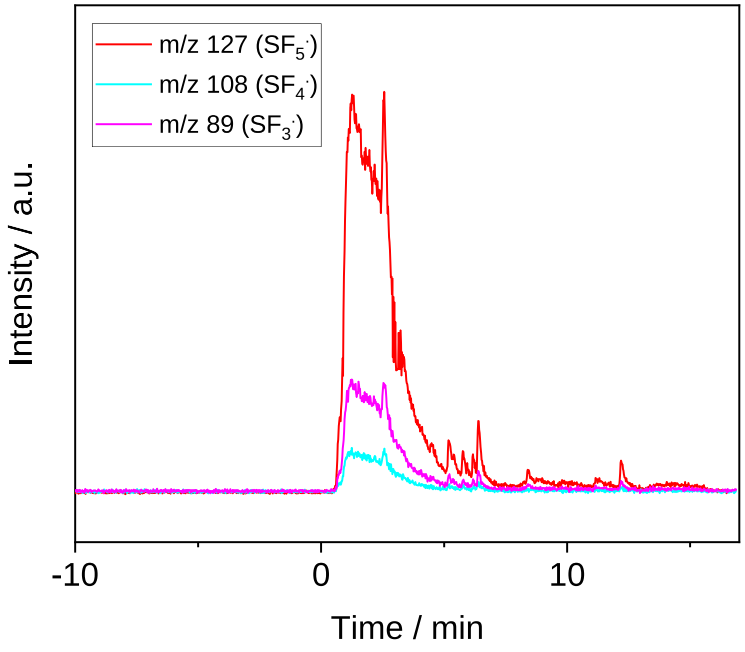

Figure S12: Acid-induced gas release from MFU-4 loaded with SF_6 (Sample 3c) followed by mass spectrometry (EI). The time point “0 min” corresponds to the addition of concentrated sulfuric acid. Sharp signals around the time point “6 min” correspond to the release of small visible gas bubbles (which were dispersed in the reaction solution) after manually shaking the reaction vessel.
Signals detected (*m*/*z*): 127 ($SF_5^{\cdot}$), 108 ($SF_4^{\cdot}$) and 89 ($SF_3^{\cdot}$).

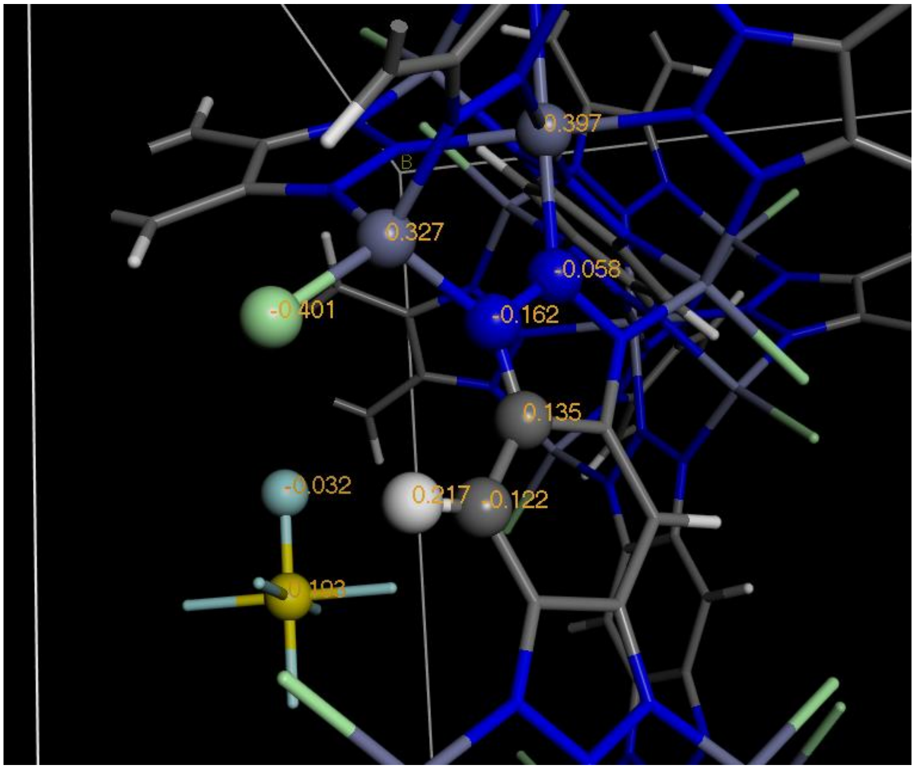

Figure S13: Electrostatic potential-derived partial (ESP) charges for MFU-4 and for SF_6. Symmetry unique atoms are high-lighted as spheres. Exact values for charges are available from the exemplary GULP input file shown below.

GULP input file for a linear transition scan of SF_6 through the narrow pore of MFU-4:

```
opti conse qok nomod pres spat noauto
ftol 1e-005
gtol 0.0001
xtol 1e-005
maxcyc 4000

title
GULP calculation from Materials Studio for MFU-4-cryst-SF6
end


cell
21.697000 21.697000 21.697000 90.000000 90.000000 90.000000 0 0 0 0 0 0

fractional
N1 core 0.714238 0.214238 0.883241 -0.161826 1.000000 0.0 1 1 1
C1 core 0.727417 0.227417 0.943692 0.135252 1.000000 0.0 1 1 1
N1 core 0.214238 0.116759 0.285762 -0.161826 1.000000 0.0 1 1 1
C1 core 0.227417 0.056308 0.272583 0.135252 1.000000 0.0 1 1 1
N1 core 0.714238 0.616759 0.285762 -0.161826 1.000000 0.0 1 1 1
C1 core 0.727417 0.556308 0.272583 0.135252 1.000000 0.0 1 1 1
N1 core 0.116759 0.714238 0.785762 -0.161826 1.000000 0.0 1 1 1
C1 core 0.056308 0.727417 0.772583 0.135252 1.000000 0.0 1 1 1
N1 core 0.616759 0.214238 0.785762 -0.161826 1.000000 0.0 1 1 1
C1 core 0.556308 0.227417 0.772583 0.135252 1.000000 0.0 1 1 1
Zn1 core 0.161331 0.661331 0.838669 0.327035 1.000000 0.0 1 1 1
Cl1 core 0.103660 0.603660 0.896340 -0.400864 1.000000 0.0 1 1 1
N1 core 0.250000 0.152222 0.250000 -0.058241 1.000000 0.0 1 1 1
N1 core 0.750000 0.652222 0.250000 -0.058241 1.000000 0.0 1 1 1
N1 core 0.285762 0.785762 0.883241 -0.161826 1.000000 0.0 1 1 1
C1 core 0.272583 0.772583 0.943692 0.135252 1.000000 0.0 1 1 1
N1 core 0.785762 0.883241 0.285762 -0.161826 1.000000 0.0 1 1 1
C1 core 0.772583 0.943692 0.272583 0.135252 1.000000 0.0 1 1 1
N1 core 0.285762 0.383241 0.285762 -0.161826 1.000000 0.0 1 1 1
C1 core 0.272583 0.443692 0.272583 0.135252 1.000000 0.0 1 1 1
N1 core 0.883241 0.285762 0.785762 -0.161826 1.000000 0.0 1 1 1
C1 core 0.943692 0.272583 0.772583 0.135252 1.000000 0.0 1 1 1
N1 core 0.383241 0.785762 0.785762 -0.161826 1.000000 0.0 1 1 1
C1 core 0.443692 0.772583 0.772583 0.135252 1.000000 0.0 1 1 1
Zn1 core 0.838669 0.338669 0.838669 0.327035 1.000000 0.0 1 1 1
Cl1 core 0.896340 0.396340 0.896340 -0.400864 1.000000 0.0 1 1 1
N1 core 0.750000 0.847778 0.250000 -0.058241 1.000000 0.0 1 1 1
N1 core 0.250000 0.347778 0.250000 -0.058241 1.000000 0.0 1 1 1
N1 core 0.785762 0.714238 0.883241 -0.161826 1.000000 0.0 1 1 1
C1 core 0.772583 0.727417 0.943692 0.135252 1.000000 0.0 1 1 1
N1 core 0.883241 0.214238 0.285762 -0.161826 1.000000 0.0 1 1 1
C1 core 0.943692 0.227417 0.272583 0.135252 1.000000 0.0 1 1 1
N1 core 0.383241 0.714238 0.285762 -0.161826 1.000000 0.0 1 1 1
C1 core 0.443692 0.727417 0.272583 0.135252 1.000000 0.0 1 1 1
N1 core 0.285762 0.116759 0.785762 -0.161826 1.000000 0.0 1 1 1
C1 core 0.272583 0.056308 0.772583 0.135252 1.000000 0.0 1 1 1
N1 core 0.785762 0.616759 0.785762 -0.161826 1.000000 0.0 1 1 1
C1 core 0.772583 0.556308 0.772583 0.135252 1.000000 0.0 1 1 1
Zn1 core 0.338669 0.161331 0.838669 0.327035 1.000000 0.0 1 1 1
Cl1 core 0.396340 0.103660 0.896340 -0.400864 1.000000 0.0 1 1 1
N1 core 0.847778 0.250000 0.250000 -0.058241 1.000000 0.0 1 1 1
N1 core 0.347778 0.750000 0.250000 -0.058241 1.000000 0.0 1 1 1
N1 core 0.214238 0.285762 0.883241 -0.161826 1.000000 0.0 1 1 1
C1 core 0.227417 0.272583 0.943692 0.135252 1.000000 0.0 1 1 1
N1 core 0.116759 0.785762 0.285762 -0.161826 1.000000 0.0 1 1 1
C1 core 0.056308 0.772583 0.272583 0.135252 1.000000 0.0 1 1 1
N1 core 0.616759 0.285762 0.285762 -0.161826 1.000000 0.0 1 1 1
C1 core 0.556308 0.272583 0.272583 0.135252 1.000000 0.0 1 1 1
```

N1 core 0.714238 0.883241 0.785762 -0.161826 1.000000 0.0 1 1 1
C1 core 0.727417 0.943692 0.772583 0.135252 1.000000 0.0 1 1 1
N1 core 0.214238 0.383241 0.785762 -0.161826 1.000000 0.0 1 1 1
C1 core 0.227417 0.443692 0.772583 0.135252 1.000000 0.0 1 1 1
Zn1 core 0.661331 0.838669 0.838669 0.327035 1.000000 0.0 1 1 1
Cl1 core 0.603660 0.896340 0.896340 -0.400864 1.000000 0.0 1 1 1
N1 core 0.152222 0.750000 0.250000 -0.058241 1.000000 0.0 1 1 1
N1 core 0.652222 0.250000 0.250000 -0.058241 1.000000 0.0 1 1 1
N1 core 0.285762 0.214238 0.116759 -0.161826 1.000000 0.0 1 1 1
C1 core 0.272583 0.227417 0.056308 0.135252 1.000000 0.0 1 1 1
N1 core 0.785762 0.116759 0.714238 -0.161826 1.000000 0.0 1 1 1
C1 core 0.772583 0.056308 0.727417 0.135252 1.000000 0.0 1 1 1
N1 core 0.285762 0.616759 0.714238 -0.161826 1.000000 0.0 1 1 1
C1 core 0.272583 0.556308 0.727417 0.135252 1.000000 0.0 1 1 1
N1 core 0.883241 0.714238 0.214238 -0.161826 1.000000 0.0 1 1 1
C1 core 0.943692 0.727417 0.227417 0.135252 1.000000 0.0 1 1 1
N1 core 0.383241 0.214238 0.214238 -0.161826 1.000000 0.0 1 1 1
C1 core 0.443692 0.227417 0.227417 0.135252 1.000000 0.0 1 1 1
Zn1 core 0.838669 0.661331 0.161331 0.327035 1.000000 0.0 1 1 1
Cl1 core 0.896340 0.603660 0.103660 -0.400864 1.000000 0.0 1 1 1
N1 core 0.750000 0.152222 0.750000 -0.058241 1.000000 0.0 1 1 1
N1 core 0.250000 0.652222 0.750000 -0.058241 1.000000 0.0 1 1 1
N1 core 0.714238 0.785762 0.116759 -0.161826 1.000000 0.0 1 1 1
C1 core 0.727417 0.772583 0.056308 0.135252 1.000000 0.0 1 1 1
N1 core 0.214238 0.883241 0.714238 -0.161826 1.000000 0.0 1 1 1
C1 core 0.227417 0.943692 0.727417 0.135252 1.000000 0.0 1 1 1
N1 core 0.714238 0.383241 0.714238 -0.161826 1.000000 0.0 1 1 1
C1 core 0.727417 0.443692 0.727417 0.135252 1.000000 0.0 1 1 1
N1 core 0.116759 0.285762 0.214238 -0.161826 1.000000 0.0 1 1 1
C1 core 0.056308 0.272583 0.227417 0.135252 1.000000 0.0 1 1 1
N1 core 0.616759 0.785762 0.214238 -0.161826 1.000000 0.0 1 1 1
C1 core 0.556308 0.772583 0.227417 0.135252 1.000000 0.0 1 1 1
Zn1 core 0.161331 0.338669 0.161331 0.327035 1.000000 0.0 1 1 1
Cl1 core 0.103660 0.396340 0.103660 -0.400864 1.000000 0.0 1 1 1
N1 core 0.250000 0.847778 0.750000 -0.058241 1.000000 0.0 1 1 1
N1 core 0.750000 0.347778 0.750000 -0.058241 1.000000 0.0 1 1 1
N1 core 0.214238 0.714238 0.116759 -0.161826 1.000000 0.0 1 1 1
C1 core 0.227417 0.727417 0.056308 0.135252 1.000000 0.0 1 1 1
N1 core 0.116759 0.214238 0.714238 -0.161826 1.000000 0.0 1 1 1
C1 core 0.056308 0.227417 0.727417 0.135252 1.000000 0.0 1 1 1
N1 core 0.616759 0.714238 0.714238 -0.161826 1.000000 0.0 1 1 1
C1 core 0.556308 0.727417 0.727417 0.135252 1.000000 0.0 1 1 1
N1 core 0.714238 0.116759 0.214238 -0.161826 1.000000 0.0 1 1 1
C1 core 0.727417 0.056308 0.227417 0.135252 1.000000 0.0 1 1 1
N1 core 0.214238 0.616759 0.214238 -0.161826 1.000000 0.0 1 1 1
C1 core 0.227417 0.556308 0.227417 0.135252 1.000000 0.0 1 1 1
Zn1 core 0.661331 0.161331 0.161331 0.327035 1.000000 0.0 1 1 1
Cl1 core 0.603660 0.103660 0.103660 -0.400864 1.000000 0.0 1 1 1
N1 core 0.152222 0.250000 0.750000 -0.058241 1.000000 0.0 1 1 1
N1 core 0.652222 0.750000 0.750000 -0.058241 1.000000 0.0 1 1 1
N1 core 0.785762 0.285762 0.116759 -0.161826 1.000000 0.0 1 1 1
C1 core 0.772583 0.272583 0.056308 0.135252 1.000000 0.0 1 1 1
N1 core 0.883241 0.785762 0.714238 -0.161826 1.000000 0.0 1 1 1
C1 core 0.943692 0.772583 0.727417 0.135252 1.000000 0.0 1 1 1
N1 core 0.383241 0.285762 0.714238 -0.161826 1.000000 0.0 1 1 1
C1 core 0.443692 0.272583 0.727417 0.135252 1.000000 0.0 1 1 1
N1 core 0.285762 0.883241 0.214238 -0.161826 1.000000 0.0 1 1 1
C1 core 0.272583 0.943692 0.227417 0.135252 1.000000 0.0 1 1 1
N1 core 0.785762 0.383241 0.214238 -0.161826 1.000000 0.0 1 1 1
C1 core 0.772583 0.443692 0.227417 0.135252 1.000000 0.0 1 1 1
Zn1 core 0.338669 0.838669 0.161331 0.327035 1.000000 0.0 1 1 1
Cl1 core 0.396340 0.896340 0.103660 -0.400864 1.000000 0.0 1 1 1
N1 core 0.847778 0.750000 0.750000 -0.058241 1.000000 0.0 1 1 1
N1 core 0.347778 0.250000 0.750000 -0.058241 1.000000 0.0 1 1 1
N1 core 0.285762 0.785762 0.116759 -0.161826 1.000000 0.0 1 1 1

```
C1 core 0.272583 0.772583 0.056308 0.135252 1.000000 0.0 1 1 1
N1 core 0.785762 0.883241 0.714238 -0.161826 1.000000 0.0 1 1 1
C1 core 0.772583 0.943692 0.727417 0.135252 1.000000 0.0 1 1 1
N1 core 0.285762 0.383241 0.714238 -0.161826 1.000000 0.0 1 1 1
C1 core 0.272583 0.443692 0.727417 0.135252 1.000000 0.0 1 1 1
N1 core 0.883241 0.285762 0.214238 -0.161826 1.000000 0.0 1 1 1
C1 core 0.943692 0.272583 0.227417 0.135252 1.000000 0.0 1 1 1
N1 core 0.383241 0.785762 0.214238 -0.161826 1.000000 0.0 1 1 1
C1 core 0.443692 0.772583 0.227417 0.135252 1.000000 0.0 1 1 1
Zn1 core 0.838669 0.338669 0.161331 0.327035 1.000000 0.0 1 1 1
Cl1 core 0.896340 0.396340 0.103660 -0.400864 1.000000 0.0 1 1 1
N1 core 0.750000 0.847778 0.750000 -0.058241 1.000000 0.0 1 1 1
N1 core 0.250000 0.347778 0.750000 -0.058241 1.000000 0.0 1 1 1
N1 core 0.714238 0.214238 0.116759 -0.161826 1.000000 0.0 1 1 1
C1 core 0.727417 0.227417 0.056308 0.135252 1.000000 0.0 1 1 1
N1 core 0.214238 0.116759 0.714238 -0.161826 1.000000 0.0 1 1 1
C1 core 0.227417 0.056308 0.727417 0.135252 1.000000 0.0 1 1 1
N1 core 0.714238 0.616759 0.714238 -0.161826 1.000000 0.0 1 1 1
C1 core 0.727417 0.556308 0.727417 0.135252 1.000000 0.0 1 1 1
N1 core 0.116759 0.714238 0.214238 -0.161826 1.000000 0.0 1 1 1
C1 core 0.056308 0.727417 0.227417 0.135252 1.000000 0.0 1 1 1
N1 core 0.616759 0.214238 0.214238 -0.161826 1.000000 0.0 1 1 1
C1 core 0.556308 0.227417 0.227417 0.135252 1.000000 0.0 1 1 1
Zn1 core 0.161331 0.661331 0.161331 0.327035 1.000000 0.0 1 1 1
Cl1 core 0.103660 0.603660 0.103660 -0.400864 1.000000 0.0 1 1 1
N1 core 0.250000 0.152222 0.750000 -0.058241 1.000000 0.0 1 1 1
N1 core 0.750000 0.652222 0.750000 -0.058241 1.000000 0.0 1 1 1
N1 core 0.214238 0.285762 0.116759 -0.161826 1.000000 0.0 1 1 1
C1 core 0.227417 0.272583 0.056308 0.135252 1.000000 0.0 1 1 1
N1 core 0.116759 0.785762 0.714238 -0.161826 1.000000 0.0 1 1 1
C1 core 0.056308 0.772583 0.727417 0.135252 1.000000 0.0 1 1 1
N1 core 0.616759 0.285762 0.714238 -0.161826 1.000000 0.0 1 1 1
C1 core 0.556308 0.272583 0.727417 0.135252 1.000000 0.0 1 1 1
N1 core 0.714238 0.883241 0.214238 -0.161826 1.000000 0.0 1 1 1
C1 core 0.727417 0.943692 0.227417 0.135252 1.000000 0.0 1 1 1
N1 core 0.214238 0.383241 0.214238 -0.161826 1.000000 0.0 1 1 1
C1 core 0.227417 0.443692 0.227417 0.135252 1.000000 0.0 1 1 1
Zn1 core 0.661331 0.838669 0.161331 0.327035 1.000000 0.0 1 1 1
Cl1 core 0.603660 0.896340 0.103660 -0.400864 1.000000 0.0 1 1 1
N1 core 0.152222 0.750000 0.750000 -0.058241 1.000000 0.0 1 1 1
N1 core 0.652222 0.250000 0.750000 -0.058241 1.000000 0.0 1 1 1
N1 core 0.785762 0.714238 0.116759 -0.161826 1.000000 0.0 1 1 1
C1 core 0.772583 0.727417 0.056308 0.135252 1.000000 0.0 1 1 1
N1 core 0.883241 0.214238 0.714238 -0.161826 1.000000 0.0 1 1 1
C1 core 0.943692 0.227417 0.727417 0.135252 1.000000 0.0 1 1 1
N1 core 0.383241 0.714238 0.714238 -0.161826 1.000000 0.0 1 1 1
C1 core 0.443692 0.727417 0.727417 0.135252 1.000000 0.0 1 1 1
N1 core 0.285762 0.116759 0.214238 -0.161826 1.000000 0.0 1 1 1
C1 core 0.272583 0.056308 0.227417 0.135252 1.000000 0.0 1 1 1
N1 core 0.785762 0.616759 0.214238 -0.161826 1.000000 0.0 1 1 1
C1 core 0.772583 0.556308 0.227417 0.135252 1.000000 0.0 1 1 1
Zn1 core 0.338669 0.161331 0.161331 0.327035 1.000000 0.0 1 1 1
Cl1 core 0.396340 0.103660 0.103660 -0.400864 1.000000 0.0 1 1 1
N1 core 0.847778 0.250000 0.750000 -0.058241 1.000000 0.0 1 1 1
N1 core 0.347778 0.750000 0.750000 -0.058241 1.000000 0.0 1 1 1
N1 core 0.714238 0.785762 0.883241 -0.161826 1.000000 0.0 1 1 1
C1 core 0.727417 0.772583 0.943692 0.135252 1.000000 0.0 1 1 1
N1 core 0.214238 0.883241 0.285762 -0.161826 1.000000 0.0 1 1 1
C1 core 0.227417 0.943692 0.272583 0.135252 1.000000 0.0 1 1 1
N1 core 0.714238 0.383241 0.285762 -0.161826 1.000000 0.0 1 1 1
C1 core 0.727417 0.443692 0.272583 0.135252 1.000000 0.0 1 1 1
N1 core 0.116759 0.285762 0.785762 -0.161826 1.000000 0.0 1 1 1
C1 core 0.056308 0.272583 0.772583 0.135252 1.000000 0.0 1 1 1
N1 core 0.616759 0.785762 0.785762 -0.161826 1.000000 0.0 1 1 1
C1 core 0.556308 0.772583 0.772583 0.135252 1.000000 0.0 1 1 1
```

```
Zn1 core 0.161331 0.338669 0.838669 0.327035 1.000000 0.0 1 1 1
Cl1 core 0.103660 0.396340 0.896340 -0.400864 1.000000 0.0 1 1 1
N1 core 0.250000 0.847778 0.250000 -0.058241 1.000000 0.0 1 1 1
N1 core 0.750000 0.347778 0.250000 -0.058241 1.000000 0.0 1 1 1
N1 core 0.285762 0.214238 0.883241 -0.161826 1.000000 0.0 1 1 1
C1 core 0.272583 0.227417 0.943692 0.135252 1.000000 0.0 1 1 1
N1 core 0.785762 0.116759 0.285762 -0.161826 1.000000 0.0 1 1 1
C1 core 0.772583 0.056308 0.272583 0.135252 1.000000 0.0 1 1 1
N1 core 0.285762 0.616759 0.285762 -0.161826 1.000000 0.0 1 1 1
C1 core 0.272583 0.556308 0.272583 0.135252 1.000000 0.0 1 1 1
N1 core 0.883241 0.714238 0.785762 -0.161826 1.000000 0.0 1 1 1
C1 core 0.943692 0.727417 0.772583 0.135252 1.000000 0.0 1 1 1
N1 core 0.383241 0.214238 0.785762 -0.161826 1.000000 0.0 1 1 1
C1 core 0.443692 0.227417 0.772583 0.135252 1.000000 0.0 1 1 1
Zn1 core 0.838669 0.661331 0.838669 0.327035 1.000000 0.0 1 1 1
Cl1 core 0.896340 0.603660 0.896340 -0.400864 1.000000 0.0 1 1 1
N1 core 0.750000 0.152222 0.250000 -0.058241 1.000000 0.0 1 1 1
N1 core 0.250000 0.652222 0.250000 -0.058241 1.000000 0.0 1 1 1
N1 core 0.785762 0.285762 0.883241 -0.161826 1.000000 0.0 1 1 1
C1 core 0.772583 0.272583 0.943692 0.135252 1.000000 0.0 1 1 1
N1 core 0.883241 0.785762 0.285762 -0.161826 1.000000 0.0 1 1 1
C1 core 0.943692 0.772583 0.272583 0.135252 1.000000 0.0 1 1 1
N1 core 0.383241 0.285762 0.285762 -0.161826 1.000000 0.0 1 1 1
C1 core 0.443692 0.272583 0.272583 0.135252 1.000000 0.0 1 1 1
N1 core 0.285762 0.883241 0.785762 -0.161826 1.000000 0.0 1 1 1
C1 core 0.272583 0.943692 0.772583 0.135252 1.000000 0.0 1 1 1
N1 core 0.785762 0.383241 0.785762 -0.161826 1.000000 0.0 1 1 1
C1 core 0.772583 0.443692 0.772583 0.135252 1.000000 0.0 1 1 1
Zn1 core 0.338669 0.838669 0.838669 0.327035 1.000000 0.0 1 1 1
Cl1 core 0.396340 0.896340 0.896340 -0.400864 1.000000 0.0 1 1 1
N1 core 0.847778 0.750000 0.250000 -0.058241 1.000000 0.0 1 1 1
N1 core 0.347778 0.250000 0.250000 -0.058241 1.000000 0.0 1 1 1
N1 core 0.214238 0.714238 0.883241 -0.161826 1.000000 0.0 1 1 1
C1 core 0.227417 0.727417 0.943692 0.135252 1.000000 0.0 1 1 1
N1 core 0.116759 0.214238 0.285762 -0.161826 1.000000 0.0 1 1 1
C1 core 0.056308 0.227417 0.272583 0.135252 1.000000 0.0 1 1 1
N1 core 0.616759 0.714238 0.285762 -0.161826 1.000000 0.0 1 1 1
C1 core 0.556308 0.727417 0.272583 0.135252 1.000000 0.0 1 1 1
N1 core 0.714238 0.116759 0.785762 -0.161826 1.000000 0.0 1 1 1
C1 core 0.727417 0.056308 0.772583 0.135252 1.000000 0.0 1 1 1
N1 core 0.214238 0.616759 0.785762 -0.161826 1.000000 0.0 1 1 1
C1 core 0.227417 0.556308 0.772583 0.135252 1.000000 0.0 1 1 1
Zn1 core 0.661331 0.161331 0.838669 0.327035 1.000000 0.0 1 1 1
Cl1 core 0.603660 0.103660 0.896340 -0.400864 1.000000 0.0 1 1 1
N1 core 0.152222 0.250000 0.250000 -0.058241 1.000000 0.0 1 1 1
N1 core 0.652222 0.750000 0.250000 -0.058241 1.000000 0.0 1 1 1
N1 core 0.214238 0.214238 0.383241 -0.161826 1.000000 0.0 1 1 1
C1 core 0.227417 0.227417 0.443692 0.135252 1.000000 0.0 1 1 1
Zn1 core 0.661331 0.661331 0.338669 0.327035 1.000000 0.0 1 1 1
Cl1 core 0.603660 0.603660 0.396340 -0.400864 1.000000 0.0 1 1 1
Zn1 core 0.161331 0.161331 0.338669 0.327035 1.000000 0.0 1 1 1
Cl1 core 0.103660 0.103660 0.396340 -0.400864 1.000000 0.0 1 1 1
N1 core 0.250000 0.250000 0.347778 -0.058241 1.000000 0.0 1 1 1
N1 core 0.785762 0.785762 0.383241 -0.161826 1.000000 0.0 1 1 1
C1 core 0.772583 0.772583 0.443692 0.135252 1.000000 0.0 1 1 1
Zn1 core 0.338669 0.338669 0.338669 0.327035 1.000000 0.0 1 1 1
Cl1 core 0.396340 0.396340 0.396340 -0.400864 1.000000 0.0 1 1 1
Zn1 core 0.838669 0.838669 0.338669 0.327035 1.000000 0.0 1 1 1
Cl1 core 0.896340 0.896340 0.396340 -0.400864 1.000000 0.0 1 1 1
N1 core 0.750000 0.750000 0.347778 -0.058241 1.000000 0.0 1 1 1
N1 core 0.785762 0.214238 0.383241 -0.161826 1.000000 0.0 1 1 1
C1 core 0.772583 0.227417 0.443692 0.135252 1.000000 0.0 1 1 1
Zn1 core 0.338669 0.661331 0.338669 0.327035 1.000000 0.0 1 1 1
Cl1 core 0.396340 0.603660 0.396340 -0.400864 1.000000 0.0 1 1 1
Zn1 core 0.838669 0.161331 0.338669 0.327035 1.000000 0.0 1 1 1
```

```
Cl1 core 0.896340 0.103660 0.396340 -0.400864 1.000000 0.0 1 1 1
N1 core 0.750000 0.250000 0.347778 -0.058241 1.000000 0.0 1 1 1
N1 core 0.214238 0.785762 0.383241 -0.161826 1.000000 0.0 1 1 1
C1 core 0.227417 0.772583 0.443692 0.135252 1.000000 0.0 1 1 1
Zn1 core 0.661331 0.338669 0.338669 0.327035 1.000000 0.0 1 1 1
Cl1 core 0.603660 0.396340 0.396340 -0.400864 1.000000 0.0 1 1 1
Zn1 core 0.161331 0.838669 0.338669 0.327035 1.000000 0.0 1 1 1
Cl1 core 0.103660 0.896340 0.396340 -0.400864 1.000000 0.0 1 1 1
N1 core 0.250000 0.750000 0.347778 -0.058241 1.000000 0.0 1 1 1
N1 core 0.785762 0.214238 0.616759 -0.161826 1.000000 0.0 1 1 1
C1 core 0.772583 0.227417 0.556308 0.135252 1.000000 0.0 1 1 1
Zn1 core 0.338669 0.661331 0.661331 0.327035 1.000000 0.0 1 1 1
Cl1 core 0.396340 0.603660 0.603660 -0.400864 1.000000 0.0 1 1 1
Zn1 core 0.838669 0.161331 0.661331 0.327035 1.000000 0.0 1 1 1
Cl1 core 0.896340 0.103660 0.603660 -0.400864 1.000000 0.0 1 1 1
N1 core 0.750000 0.250000 0.652222 -0.058241 1.000000 0.0 1 1 1
N1 core 0.214238 0.785762 0.616759 -0.161826 1.000000 0.0 1 1 1
C1 core 0.227417 0.772583 0.556308 0.135252 1.000000 0.0 1 1 1
Zn1 core 0.661331 0.338669 0.661331 0.327035 1.000000 0.0 1 1 1
Cl1 core 0.603660 0.396340 0.603660 -0.400864 1.000000 0.0 1 1 1
Zn1 core 0.161331 0.838669 0.661331 0.327035 1.000000 0.0 1 1 1
Cl1 core 0.103660 0.896340 0.603660 -0.400864 1.000000 0.0 1 1 1
N1 core 0.250000 0.750000 0.652222 -0.058241 1.000000 0.0 1 1 1
N1 core 0.214238 0.214238 0.616759 -0.161826 1.000000 0.0 1 1 1
C1 core 0.227417 0.227417 0.556308 0.135252 1.000000 0.0 1 1 1
Zn1 core 0.661331 0.661331 0.661331 0.327035 1.000000 0.0 1 1 1
Cl1 core 0.603660 0.603660 0.603660 -0.400864 1.000000 0.0 1 1 1
Zn1 core 0.161331 0.161331 0.661331 0.327035 1.000000 0.0 1 1 1
Cl1 core 0.103660 0.103660 0.603660 -0.400864 1.000000 0.0 1 1 1
N1 core 0.250000 0.250000 0.652222 -0.058241 1.000000 0.0 1 1 1
N1 core 0.785762 0.785762 0.616759 -0.161826 1.000000 0.0 1 1 1
C1 core 0.772583 0.772583 0.556308 0.135252 1.000000 0.0 1 1 1
Zn1 core 0.338669 0.338669 0.661331 0.327035 1.000000 0.0 1 1 1
Cl1 core 0.396340 0.396340 0.603660 -0.400864 1.000000 0.0 1 1 1
Zn1 core 0.838669 0.838669 0.661331 0.327035 1.000000 0.0 1 1 1
Cl1 core 0.896340 0.896340 0.603660 -0.400864 1.000000 0.0 1 1 1
N1 core 0.750000 0.750000 0.652222 -0.058241 1.000000 0.0 1 1 1
N1 core 0.285762 0.714238 0.383241 -0.161826 1.000000 0.0 1 1 1
C1 core 0.272583 0.727417 0.443692 0.135252 1.000000 0.0 1 1 1
Zn1 core 0.750000 0.250000 0.750000 0.397367 1.000000 0.0 1 1 1
N1 core 0.750000 0.250000 0.847778 -0.058241 1.000000 0.0 1 1 1
N1 core 0.714238 0.285762 0.383241 -0.161826 1.000000 0.0 1 1 1
C1 core 0.727417 0.272583 0.443692 0.135252 1.000000 0.0 1 1 1
Zn1 core 0.250000 0.750000 0.750000 0.397367 1.000000 0.0 1 1 1
N1 core 0.250000 0.750000 0.847778 -0.058241 1.000000 0.0 1 1 1
N1 core 0.285762 0.285762 0.383241 -0.161826 1.000000 0.0 1 1 1
C1 core 0.272583 0.272583 0.443692 0.135252 1.000000 0.0 1 1 1
Zn1 core 0.750000 0.750000 0.750000 0.397367 1.000000 0.0 1 1 1
N1 core 0.750000 0.750000 0.847778 -0.058241 1.000000 0.0 1 1 1
N1 core 0.714238 0.714238 0.383241 -0.161826 1.000000 0.0 1 1 1
C1 core 0.727417 0.727417 0.443692 0.135252 1.000000 0.0 1 1 1
Zn1 core 0.250000 0.250000 0.750000 0.397367 1.000000 0.0 1 1 1
N1 core 0.250000 0.250000 0.847778 -0.058241 1.000000 0.0 1 1 1
N1 core 0.714238 0.714238 0.616759 -0.161826 1.000000 0.0 1 1 1
C1 core 0.727417 0.727417 0.556308 0.135252 1.000000 0.0 1 1 1
Zn1 core 0.250000 0.250000 0.250000 0.397367 1.000000 0.0 1 1 1
N1 core 0.250000 0.250000 0.152222 -0.058241 1.000000 0.0 1 1 1
N1 core 0.285762 0.285762 0.616759 -0.161826 1.000000 0.0 1 1 1
C1 core 0.272583 0.272583 0.556308 0.135252 1.000000 0.0 1 1 1
Zn1 core 0.750000 0.750000 0.250000 0.397367 1.000000 0.0 1 1 1
N1 core 0.750000 0.750000 0.152222 -0.058241 1.000000 0.0 1 1 1
N1 core 0.714238 0.285762 0.616759 -0.161826 1.000000 0.0 1 1 1
C1 core 0.727417 0.272583 0.556308 0.135252 1.000000 0.0 1 1 1
Zn1 core 0.250000 0.750000 0.250000 0.397367 1.000000 0.0 1 1 1
N1 core 0.250000 0.750000 0.152222 -0.058241 1.000000 0.0 1 1 1
```

```
N1 core 0.285762 0.714238 0.616759 -0.161826 1.000000 0.0 1 1 1
C1 core 0.272583 0.727417 0.556308 0.135252 1.000000 0.0 1 1 1
Zn1 core 0.750000 0.250000 0.250000 0.397367 1.000000 0.0 1 1 1
N1 core 0.750000 0.250000 0.152222 -0.058241 1.000000 0.0 1 1 1
C1 core 0.702883 0.702883 0.500000 -0.122151 1.000000 0.0 1 1 1
H1 core 0.667536 0.667536 0.500000 0.216532 1.000000 0.0 1 1 1
C1 core 0.202883 0.202883 0.500000 -0.122151 1.000000 0.0 1 1 1
H1 core 0.167536 0.167536 0.500000 0.216532 1.000000 0.0 1 1 1
C1 core 0.297117 0.297117 0.500000 -0.122151 1.000000 0.0 1 1 1
H1 core 0.332464 0.332464 0.500000 0.216532 1.000000 0.0 1 1 1
C1 core 0.797117 0.797117 0.500000 -0.122151 1.000000 0.0 1 1 1
H1 core 0.832464 0.832464 0.500000 0.216532 1.000000 0.0 1 1 1
C1 core 0.297117 0.702883 0.500000 -0.122151 1.000000 0.0 1 1 1
H1 core 0.332464 0.667536 0.500000 0.216532 1.000000 0.0 1 1 1
C1 core 0.797117 0.202883 0.500000 -0.122151 1.000000 0.0 1 1 1
H1 core 0.832464 0.167536 0.500000 0.216532 1.000000 0.0 1 1 1
C1 core 0.702883 0.297117 0.500000 -0.122151 1.000000 0.0 1 1 1
H1 core 0.667536 0.332464 0.500000 0.216532 1.000000 0.0 1 1 1
C1 core 0.202883 0.797117 0.500000 -0.122151 1.000000 0.0 1 1 1
H1 core 0.167536 0.832464 0.500000 0.216532 1.000000 0.0 1 1 1
C1 core 0.202883 0.702883 0.000000 -0.122151 1.000000 0.0 1 1 1
H1 core 0.167536 0.667536 0.000000 0.216532 1.000000 0.0 1 1 1
C1 core 0.797117 0.297117 0.000000 -0.122151 1.000000 0.0 1 1 1
H1 core 0.832464 0.332464 0.000000 0.216532 1.000000 0.0 1 1 1
C1 core 0.297117 0.202883 0.000000 -0.122151 1.000000 0.0 1 1 1
H1 core 0.332464 0.167536 0.000000 0.216532 1.000000 0.0 0 0 0
C1 core 0.702883 0.797117 0.000000 -0.122151 1.000000 0.0 1 1 1
H1 core 0.667536 0.832464 0.000000 0.216532 1.000000 0.0 0 0 0
C1 core 0.797117 0.702883 0.000000 -0.122151 1.000000 0.0 1 1 1
H1 core 0.832464 0.667536 0.000000 0.216532 1.000000 0.0 1 1 1
C1 core 0.202883 0.297117 0.000000 -0.122151 1.000000 0.0 1 1 1
H1 core 0.167536 0.332464 0.000000 0.216532 1.000000 0.0 1 1 1
C1 core 0.702883 0.202883 0.000000 -0.122151 1.000000 0.0 1 1 1
H1 core 0.667536 0.167536 0.000000 0.216532 1.000000 0.0 0 0 0
C1 core 0.297117 0.797117 0.000000 -0.122151 1.000000 0.0 1 1 1
H1 core 0.332464 0.832464 0.000000 0.216532 1.000000 0.0 0 0 0
C1 core 0.702883 0.500000 0.297117 -0.122151 1.000000 0.0 1 1 1
H1 core 0.667536 0.500000 0.332464 0.216532 1.000000 0.0 1 1 1
C1 core 0.202883 0.500000 0.797117 -0.122151 1.000000 0.0 1 1 1
H1 core 0.167536 0.500000 0.832464 0.216532 1.000000 0.0 1 1 1
C1 core 0.297117 0.500000 0.297117 -0.122151 1.000000 0.0 1 1 1
H1 core 0.332464 0.500000 0.332464 0.216532 1.000000 0.0 1 1 1
C1 core 0.797117 0.500000 0.797117 -0.122151 1.000000 0.0 1 1 1
H1 core 0.832464 0.500000 0.832464 0.216532 1.000000 0.0 1 1 1
C1 core 0.500000 0.702883 0.297117 -0.122151 1.000000 0.0 1 1 1
H1 core 0.500000 0.667536 0.332464 0.216532 1.000000 0.0 1 1 1
C1 core 0.500000 0.202883 0.797117 -0.122151 1.000000 0.0 1 1 1
H1 core 0.500000 0.167536 0.832464 0.216532 1.000000 0.0 1 1 1
C1 core 0.500000 0.297117 0.297117 -0.122151 1.000000 0.0 1 1 1
H1 core 0.500000 0.332464 0.332464 0.216532 1.000000 0.0 1 1 1
C1 core 0.500000 0.797117 0.797117 -0.122151 1.000000 0.0 1 1 1
H1 core 0.500000 0.832464 0.832464 0.216532 1.000000 0.0 1 1 1
C1 core 0.297117 0.500000 0.702883 -0.122151 1.000000 0.0 1 1 1
H1 core 0.332464 0.500000 0.667536 0.216532 1.000000 0.0 1 1 1
C1 core 0.797117 0.500000 0.202883 -0.122151 1.000000 0.0 1 1 1
H1 core 0.832464 0.500000 0.167536 0.216532 1.000000 0.0 1 1 1
C1 core 0.702883 0.500000 0.702883 -0.122151 1.000000 0.0 1 1 1
H1 core 0.667536 0.500000 0.667536 0.216532 1.000000 0.0 1 1 1
C1 core 0.202883 0.500000 0.202883 -0.122151 1.000000 0.0 1 1 1
H1 core 0.167536 0.500000 0.167536 0.216532 1.000000 0.0 1 1 1
C1 core 0.500000 0.702883 0.702883 -0.122151 1.000000 0.0 1 1 1
H1 core 0.500000 0.667536 0.667536 0.216532 1.000000 0.0 1 1 1
C1 core 0.500000 0.202883 0.202883 -0.122151 1.000000 0.0 1 1 1
H1 core 0.500000 0.167536 0.167536 0.216532 1.000000 0.0 1 1 1
C1 core 0.500000 0.297117 0.702883 -0.122151 1.000000 0.0 1 1 1
```

```
H1 core 0.500000 0.332464 0.667536 0.216532 1.000000 0.0 1 1 1
C1 core 0.500000 0.797117 0.202883 -0.122151 1.000000 0.0 1 1 1
H1 core 0.500000 0.832464 0.167536 0.216532 1.000000 0.0 1 1 1
C1 core 0.202883 0.000000 0.297117 -0.122151 1.000000 0.0 1 1 1
H1 core 0.167536 0.000000 0.332464 0.216532 1.000000 0.0 1 1 1
C1 core 0.702883 0.000000 0.797117 -0.122151 1.000000 0.0 1 1 1
H1 core 0.667536 0.000000 0.832464 0.216532 1.000000 0.0 1 1 1
C1 core 0.797117 0.000000 0.297117 -0.122151 1.000000 0.0 1 1 1
H1 core 0.832464 0.000000 0.332464 0.216532 1.000000 0.0 1 1 1
C1 core 0.297117 0.000000 0.797117 -0.122151 1.000000 0.0 1 1 1
H1 core 0.332464 0.000000 0.832464 0.216532 1.000000 0.0 1 1 1
C1 core 0.000000 0.202883 0.297117 -0.122151 1.000000 0.0 1 1 1
H1 core 0.000000 0.167536 0.332464 0.216532 1.000000 0.0 1 1 1
C1 core 0.000000 0.702883 0.797117 -0.122151 1.000000 0.0 1 1 1
H1 core 0.000000 0.667536 0.832464 0.216532 1.000000 0.0 1 1 1
C1 core 0.000000 0.797117 0.297117 -0.122151 1.000000 0.0 1 1 1
H1 core 0.000000 0.832464 0.332464 0.216532 1.000000 0.0 1 1 1
C1 core 0.000000 0.297117 0.797117 -0.122151 1.000000 0.0 1 1 1
H1 core 0.000000 0.332464 0.832464 0.216532 1.000000 0.0 1 1 1
C1 core 0.797117 0.000000 0.702883 -0.122151 1.000000 0.0 1 1 1
H1 core 0.832464 0.000000 0.667536 0.216532 1.000000 0.0 1 1 1
C1 core 0.297117 0.000000 0.202883 -0.122151 1.000000 0.0 1 1 1
H1 core 0.332464 0.000000 0.167536 0.216532 1.000000 0.0 1 1 1
C1 core 0.202883 0.000000 0.702883 -0.122151 1.000000 0.0 1 1 1
H1 core 0.167536 0.000000 0.667536 0.216532 1.000000 0.0 1 1 1
C1 core 0.702883 0.000000 0.202883 -0.122151 1.000000 0.0 1 1 1
H1 core 0.667536 0.000000 0.167536 0.216532 1.000000 0.0 1 1 1
C1 core 0.000000 0.202883 0.702883 -0.122151 1.000000 0.0 1 1 1
H1 core 0.000000 0.167536 0.667536 0.216532 1.000000 0.0 1 1 1
C1 core 0.000000 0.702883 0.202883 -0.122151 1.000000 0.0 1 1 1
H1 core 0.000000 0.667536 0.167536 0.216532 1.000000 0.0 1 1 1
C1 core 0.000000 0.797117 0.702883 -0.122151 1.000000 0.0 1 1 1
H1 core 0.000000 0.832464 0.667536 0.216532 1.000000 0.0 1 1 1
C1 core 0.000000 0.297117 0.202883 -0.122151 1.000000 0.0 1 1 1
H1 core 0.000000 0.332464 0.167536 0.216532 1.000000 0.0 1 1 1
S1 core 0.500000 0.500000 0.000000 0.192857 1.000000 0.0 0 0 0 T
F1 core 0.500000 0.421165 0.000000 -0.032143 1.000000 0.0 1 1 1
F1 core 0.500000 0.578835 0.000000 -0.032143 1.000000 0.0 1 1 1
F1 core 0.578835 0.500000 0.000000 -0.032143 1.000000 0.0 1 1 1
F1 core 0.421165 0.500000 0.000000 -0.032143 1.000000 0.0 1 1 1
F1 core 0.500000 0.500000 0.078835 -0.032143 1.000000 0.0 0 0 0 T
F1 core 0.500000 0.500000 -0.078835 -0.032143 1.000000 0.0 0 0 0 T

#
# translate along c axis of the unit cell in 200 steps
#
translate 0.0 0.0 1.0 200 noise 0.05
#

Species
C1 core C_R
Cl1 core Cl
F1 core F_
H1 core H_
N1 core N_R
S1 core S_3+2
Zn1 core Zn3+2

spacegroup
P 1

connect 1 2 resonant  0 0 0
connect 1 221 single  0 0 0
connect 1 284 resonant  0 0 0
connect 2 198 resonant  0 0 0
```

```
connect 3 4 resonant  0 0 0
connect 3 13 resonant  0 0 0
connect 3 229 single  0 0 0
connect 4 162 resonant  0 0 0
connect 4 377 resonant  0 0 0
connect 5 6 resonant  0 0 0
connect 5 14 resonant  0 0 0
connect 5 227 single  0 0 0
connect 6 164 resonant  0 0 0
connect 6 345 resonant  0 0 0
connect 7 8 resonant  0 0 0
connect 7 11 single  0 0 0
connect 7 153 resonant  0 0 0
connect 8 144 resonant  0 0 0
connect 8 387 resonant  0 0 0
connect 9 10 resonant  0 0 0
connect 9 154 resonant  0 0 0
connect 9 221 single  0 0 0
connect 10 146 resonant  0 0 0
connect 10 355 resonant  0 0 0
connect 11 12 single  0 0 0
connect 11 211 single  0 0 0
connect 11 219 single  0 0 0
connect 13 161 resonant  0 0 0
connect 13 299 single  0 0 0
connect 14 163 resonant  0 0 0
connect 14 303 single  0 0 0
connect 15 16 resonant  0 0 0
connect 15 207 single  0 0 0
connect 15 288 resonant  0 0 0
connect 16 212 resonant  0 0 0
connect 17 18 resonant  0 0 0
connect 17 27 resonant  0 0 0
connect 17 236 single  0 0 0
connect 18 148 resonant  0 0 0
connect 19 20 resonant  0 0 0
connect 19 28 resonant  0 0 0
connect 19 234 single  0 0 0
connect 20 150 resonant  0 0 0
connect 20 349 resonant  0 0 0
connect 21 22 resonant  0 0 0
connect 21 25 single  0 0 0
connect 21 167 resonant  0 0 0
connect 22 158 resonant  0 0 0
connect 23 24 resonant  0 0 0
connect 23 168 resonant  0 0 0
connect 23 207 single  0 0 0
connect 24 160 resonant  0 0 0
connect 24 359 resonant  0 0 0
connect 25 26 single  0 0 0
connect 25 197 single  0 0 0
connect 25 205 single  0 0 0
connect 27 147 resonant  0 0 0
connect 27 303 single  0 0 0
connect 28 149 resonant  0 0 0
connect 28 299 single  0 0 0
connect 29 30 resonant  0 0 0
connect 29 193 single  0 0 0
connect 29 292 resonant  0 0 0
connect 30 170 resonant  0 0 0
connect 31 32 resonant  0 0 0
connect 31 41 resonant  0 0 0
connect 31 243 single  0 0 0
connect 32 120 resonant  0 0 0
connect 33 34 resonant  0 0 0
```

```
connect 33 42 resonant  0 0 0
connect 33 241 single  0 0 0
connect 34 122 resonant  0 0 0
connect 34 353 resonant  0 0 0
connect 35 36 resonant  0 0 0
connect 35 39 single  0 0 0
connect 35 139 resonant  0 0 0
connect 36 130 resonant  0 0 0
connect 36 383 resonant  0 0 0
connect 37 38 resonant  0 0 0
connect 37 140 resonant  0 0 0
connect 37 193 single  0 0 0
connect 38 132 resonant  0 0 0
connect 38 351 resonant  0 0 0
connect 39 40 single  0 0 0
connect 39 183 single  0 0 0
connect 39 191 single  0 0 0
connect 41 119 resonant  0 0 0
connect 41 311 single  0 0 0
connect 42 121 resonant  0 0 0
connect 42 307 single  0 0 0
connect 43 44 resonant  0 0 0
connect 43 179 single  0 0 0
connect 43 296 resonant  0 0 0
connect 44 184 resonant  0 0 0
connect 45 46 resonant  0 0 0
connect 45 55 resonant  0 0 0
connect 45 250 single  0 0 0
connect 46 134 resonant  0 0 0
connect 46 389 resonant  0 0 0
connect 47 48 resonant  0 0 0
connect 47 56 resonant  0 0 0
connect 47 248 single  0 0 0
connect 48 136 resonant  0 0 0
connect 48 357 resonant  0 0 0
connect 49 50 resonant  0 0 0
connect 49 53 single  0 0 0
connect 49 125 resonant  0 0 0
connect 50 116 resonant  0 0 0
connect 51 52 resonant  0 0 0
connect 51 126 resonant  0 0 0
connect 51 179 single  0 0 0
connect 52 118 resonant  0 0 0
connect 52 347 resonant  0 0 0
connect 53 54 single  0 0 0
connect 53 169 single  0 0 0
connect 53 177 single  0 0 0
connect 55 133 resonant  0 0 0
connect 55 307 single  0 0 0
connect 56 135 resonant  0 0 0
connect 56 311 single  0 0 0
connect 57 58 resonant  0 0 0
connect 57 165 single  0 0 0
connect 57 300 resonant  0 0 0
connect 58 142 resonant  0 0 0
connect 58 333 resonant  0 0 0
connect 59 60 resonant  0 0 0
connect 59 69 resonant  0 0 0
connect 59 257 single  0 0 0
connect 60 218 resonant  0 0 0
connect 60 393 resonant  0 0 0
connect 61 62 resonant  0 0 0
connect 61 70 resonant  0 0 0
connect 61 255 single  0 0 0
connect 62 220 resonant  0 0 0
```

```
connect 62 361 resonant  0 0 0
connect 63 64 resonant  0 0 0
connect 63 67 single  0 0 0
connect 63 209 resonant  0 0 0
connect 64 200 resonant  0 0 0
connect 65 66 resonant  0 0 0
connect 65 165 single  0 0 0
connect 65 210 resonant  0 0 0
connect 66 202 resonant  0 0 0
connect 66 371 resonant  0 0 0
connect 67 68 single  0 0 0
connect 67 155 single  0 0 0
connect 67 163 single  0 0 0
connect 69 217 resonant  0 0 0
connect 69 283 single  0 0 0
connect 70 219 resonant  0 0 0
connect 70 287 single  0 0 0
connect 71 72 resonant  0 0 0
connect 71 151 single  0 0 0
connect 71 304 resonant  0 0 0
connect 72 156 resonant  0 0 0
connect 72 335 resonant  0 0 0
connect 73 74 resonant  0 0 0
connect 73 83 resonant  0 0 0
connect 73 264 single  0 0 0
connect 74 204 resonant  0 0 0
connect 75 76 resonant  0 0 0
connect 75 84 resonant  0 0 0
connect 75 262 single  0 0 0
connect 76 206 resonant  0 0 0
connect 76 365 resonant  0 0 0
connect 77 78 resonant  0 0 0
connect 77 81 single  0 0 0
connect 77 223 resonant  0 0 0
connect 78 214 resonant  0 0 0
connect 78 407 resonant  0 0 0
connect 79 80 resonant  0 0 0
connect 79 151 single  0 0 0
connect 79 224 resonant  0 0 0
connect 80 216 resonant  0 0 0
connect 80 375 resonant  0 0 0
connect 81 82 single  0 0 0
connect 81 141 single  0 0 0
connect 81 149 single  0 0 0
connect 83 203 resonant  0 0 0
connect 83 287 single  0 0 0
connect 84 205 resonant  0 0 0
connect 84 283 single  0 0 0
connect 85 86 resonant  0 0 0
connect 85 137 single  0 0 0
connect 85 308 resonant  0 0 0
connect 86 114 resonant  0 0 0
connect 86 329 resonant  0 0 0
connect 87 88 resonant  0 0 0
connect 87 97 resonant  0 0 0
connect 87 271 single  0 0 0
connect 88 176 resonant  0 0 0
connect 88 401 resonant  0 0 0
connect 89 90 resonant  0 0 0
connect 89 98 resonant  0 0 0
connect 89 269 single  0 0 0
connect 90 178 resonant  0 0 0
connect 90 369 resonant  0 0 0
connect 91 92 resonant  0 0 0
connect 91 95 single  0 0 0
```

```
connect 91 195 resonant  0 0 0
connect 92 186 resonant  0 0 0
connect 92 399 resonant  0 0 0
connect 93 94 resonant  0 0 0
connect 93 137 single  0 0 0
connect 93 196 resonant  0 0 0
connect 94 188 resonant  0 0 0
connect 94 367 resonant  0 0 0
connect 95 96 single  0 0 0
connect 95 127 single  0 0 0
connect 95 135 single  0 0 0
connect 97 175 resonant  0 0 0
connect 97 295 single  0 0 0
connect 98 177 resonant  0 0 0
connect 98 291 single  0 0 0
connect 99 100 resonant  0 0 0
connect 99 123 single  0 0 0
connect 99 312 resonant  0 0 0
connect 100 128 resonant  0 0 0
connect 100 331 resonant  0 0 0
connect 101 102 resonant  0 0 0
connect 101 111 resonant  0 0 0
connect 101 278 single  0 0 0
connect 102 190 resonant  0 0 0
connect 103 104 resonant  0 0 0
connect 103 112 resonant  0 0 0
connect 103 276 single  0 0 0
connect 104 192 resonant  0 0 0
connect 104 373 resonant  0 0 0
connect 105 106 resonant  0 0 0
connect 105 109 single  0 0 0
connect 105 181 resonant  0 0 0
connect 106 172 resonant  0 0 0
connect 107 108 resonant  0 0 0
connect 107 123 single  0 0 0
connect 107 182 resonant  0 0 0
connect 108 174 resonant  0 0 0
connect 108 363 resonant  0 0 0
connect 109 110 single  0 0 0
connect 109 113 single  0 0 0
connect 109 121 single  0 0 0
connect 111 189 resonant  0 0 0
connect 111 291 single  0 0 0
connect 112 191 resonant  0 0 0
connect 112 295 single  0 0 0
connect 113 114 resonant  0 0 0
connect 113 308 resonant  0 0 0
connect 114 343 resonant  0 0 0
connect 115 116 resonant  0 0 0
connect 115 125 resonant  0 0 0
connect 115 278 single  0 0 0
connect 117 118 resonant  0 0 0
connect 117 126 resonant  0 0 0
connect 117 276 single  0 0 0
connect 118 361 resonant  0 0 0
connect 119 120 resonant  0 0 0
connect 119 123 single  0 0 0
connect 121 122 resonant  0 0 0
connect 122 375 resonant  0 0 0
connect 123 124 single  0 0 0
connect 125 291 single  0 0 0
connect 126 295 single  0 0 0
connect 127 128 resonant  0 0 0
connect 127 312 resonant  0 0 0
connect 128 341 resonant  0 0 0
```

```
connect 129 130 resonant  0 0 0
connect 129 139 resonant  0 0 0
connect 129 271 single  0 0 0
connect 130 397 resonant  0 0 0
connect 131 132 resonant  0 0 0
connect 131 140 resonant  0 0 0
connect 131 269 single  0 0 0
connect 132 365 resonant  0 0 0
connect 133 134 resonant  0 0 0
connect 133 137 single  0 0 0
connect 134 403 resonant  0 0 0
connect 135 136 resonant  0 0 0
connect 136 371 resonant  0 0 0
connect 137 138 single  0 0 0
connect 139 295 single  0 0 0
connect 140 291 single  0 0 0
connect 141 142 resonant  0 0 0
connect 141 300 resonant  0 0 0
connect 142 339 resonant  0 0 0
connect 143 144 resonant  0 0 0
connect 143 153 resonant  0 0 0
connect 143 264 single  0 0 0
connect 144 405 resonant  0 0 0
connect 145 146 resonant  0 0 0
connect 145 154 resonant  0 0 0
connect 145 262 single  0 0 0
connect 146 373 resonant  0 0 0
connect 147 148 resonant  0 0 0
connect 147 151 single  0 0 0
connect 149 150 resonant  0 0 0
connect 150 367 resonant  0 0 0
connect 151 152 single  0 0 0
connect 153 287 single  0 0 0
connect 154 283 single  0 0 0
connect 155 156 resonant  0 0 0
connect 155 304 resonant  0 0 0
connect 156 337 resonant  0 0 0
connect 157 158 resonant  0 0 0
connect 157 167 resonant  0 0 0
connect 157 257 single  0 0 0
connect 159 160 resonant  0 0 0
connect 159 168 resonant  0 0 0
connect 159 255 single  0 0 0
connect 160 369 resonant  0 0 0
connect 161 162 resonant  0 0 0
connect 161 165 single  0 0 0
connect 162 395 resonant  0 0 0
connect 163 164 resonant  0 0 0
connect 164 363 resonant  0 0 0
connect 165 166 single  0 0 0
connect 167 283 single  0 0 0
connect 168 287 single  0 0 0
connect 169 170 resonant  0 0 0
connect 169 292 resonant  0 0 0
connect 171 172 resonant  0 0 0
connect 171 181 resonant  0 0 0
connect 171 250 single  0 0 0
connect 173 174 resonant  0 0 0
connect 173 182 resonant  0 0 0
connect 173 248 single  0 0 0
connect 174 345 resonant  0 0 0
connect 175 176 resonant  0 0 0
connect 175 179 single  0 0 0
connect 176 391 resonant  0 0 0
connect 177 178 resonant  0 0 0
```

```
connect 178 359 resonant  0 0 0
connect 179 180 single  0 0 0
connect 181 307 single  0 0 0
connect 182 311 single  0 0 0
connect 183 184 resonant  0 0 0
connect 183 296 resonant  0 0 0
connect 185 186 resonant  0 0 0
connect 185 195 resonant  0 0 0
connect 185 243 single  0 0 0
connect 186 381 resonant  0 0 0
connect 187 188 resonant  0 0 0
connect 187 196 resonant  0 0 0
connect 187 241 single  0 0 0
connect 188 349 resonant  0 0 0
connect 189 190 resonant  0 0 0
connect 189 193 single  0 0 0
connect 191 192 resonant  0 0 0
connect 192 355 resonant  0 0 0
connect 193 194 single  0 0 0
connect 195 311 single  0 0 0
connect 196 307 single  0 0 0
connect 197 198 resonant  0 0 0
connect 197 284 resonant  0 0 0
connect 199 200 resonant  0 0 0
connect 199 209 resonant  0 0 0
connect 199 236 single  0 0 0
connect 201 202 resonant  0 0 0
connect 201 210 resonant  0 0 0
connect 201 234 single  0 0 0
connect 202 357 resonant  0 0 0
connect 203 204 resonant  0 0 0
connect 203 207 single  0 0 0
connect 205 206 resonant  0 0 0
connect 206 351 resonant  0 0 0
connect 207 208 single  0 0 0
connect 209 303 single  0 0 0
connect 210 299 single  0 0 0
connect 211 212 resonant  0 0 0
connect 211 288 resonant  0 0 0
connect 213 214 resonant  0 0 0
connect 213 223 resonant  0 0 0
connect 213 229 single  0 0 0
connect 214 385 resonant  0 0 0
connect 215 216 resonant  0 0 0
connect 215 224 resonant  0 0 0
connect 215 227 single  0 0 0
connect 216 353 resonant  0 0 0
connect 217 218 resonant  0 0 0
connect 217 221 single  0 0 0
connect 218 379 resonant  0 0 0
connect 219 220 resonant  0 0 0
connect 220 347 resonant  0 0 0
connect 221 222 single  0 0 0
connect 223 299 single  0 0 0
connect 224 303 single  0 0 0
connect 225 226 resonant  0 0 0
connect 225 229 single  0 0 0
connect 225 231 resonant  0 0 0
connect 226 290 resonant  0 0 0
connect 226 315 resonant  0 0 0
connect 227 228 single  0 0 0
connect 227 293 single  0 0 0
connect 229 230 single  0 0 0
connect 231 289 resonant  0 0 0
connect 231 299 single  0 0 0
```

```
connect 232 233 resonant  0 0 0
connect 232 236 single  0 0 0
connect 232 238 resonant  0 0 0
connect 233 294 resonant  0 0 0
connect 233 319 resonant  0 0 0
connect 234 235 single  0 0 0
connect 234 289 single  0 0 0
connect 236 237 single  0 0 0
connect 238 293 resonant  0 0 0
connect 238 303 single  0 0 0
connect 239 240 resonant  0 0 0
connect 239 243 single  0 0 0
connect 239 245 resonant  0 0 0
connect 240 286 resonant  0 0 0
connect 240 323 resonant  0 0 0
connect 241 242 single  0 0 0
connect 241 281 single  0 0 0
connect 243 244 single  0 0 0
connect 245 285 resonant  0 0 0
connect 245 311 single  0 0 0
connect 246 247 resonant  0 0 0
connect 246 250 single  0 0 0
connect 246 252 resonant  0 0 0
connect 247 282 resonant  0 0 0
connect 247 327 resonant  0 0 0
connect 248 249 single  0 0 0
connect 248 285 single  0 0 0
connect 250 251 single  0 0 0
connect 252 281 resonant  0 0 0
connect 252 307 single  0 0 0
connect 253 254 resonant  0 0 0
connect 253 257 single  0 0 0
connect 253 259 resonant  0 0 0
connect 254 306 resonant  0 0 0
connect 254 323 resonant  0 0 0
connect 255 256 single  0 0 0
connect 255 309 single  0 0 0
connect 257 258 single  0 0 0
connect 259 283 single  0 0 0
connect 259 305 resonant  0 0 0
connect 260 261 resonant  0 0 0
connect 260 264 single  0 0 0
connect 260 266 resonant  0 0 0
connect 261 310 resonant  0 0 0
connect 261 327 resonant  0 0 0
connect 262 263 single  0 0 0
connect 262 305 single  0 0 0
connect 264 265 single  0 0 0
connect 266 287 single  0 0 0
connect 266 309 resonant  0 0 0
connect 267 268 resonant  0 0 0
connect 267 271 single  0 0 0
connect 267 273 resonant  0 0 0
connect 268 302 resonant  0 0 0
connect 268 315 resonant  0 0 0
connect 269 270 single  0 0 0
connect 269 297 single  0 0 0
connect 271 272 single  0 0 0
connect 273 295 single  0 0 0
connect 273 301 resonant  0 0 0
connect 274 275 resonant  0 0 0
connect 274 278 single  0 0 0
connect 274 280 resonant  0 0 0
connect 275 298 resonant  0 0 0
connect 275 319 resonant  0 0 0
```

```
connect 276 277 single  0 0 0
connect 276 301 single  0 0 0
connect 278 279 single  0 0 0
connect 280 291 single  0 0 0
connect 280 297 resonant  0 0 0
connect 281 282 resonant  0 0 0
connect 282 321 resonant  0 0 0
connect 283 284 single  0 0 0
connect 285 286 resonant  0 0 0
connect 286 325 resonant  0 0 0
connect 287 288 single  0 0 0
connect 289 290 resonant  0 0 0
connect 290 317 resonant  0 0 0
connect 291 292 single  0 0 0
connect 293 294 resonant  0 0 0
connect 294 313 resonant  0 0 0
connect 295 296 single  0 0 0
connect 297 298 resonant  0 0 0
connect 298 313 resonant  0 0 0
connect 299 300 single  0 0 0
connect 301 302 resonant  0 0 0
connect 302 317 resonant  0 0 0
connect 303 304 single  0 0 0
connect 305 306 resonant  0 0 0
connect 306 325 resonant  0 0 0
connect 307 308 single  0 0 0
connect 309 310 resonant  0 0 0
connect 310 321 resonant  0 0 0
connect 311 312 single  0 0 0
connect 313 314 single  0 0 0
connect 315 316 single  0 0 0
connect 317 318 single  0 0 0
connect 319 320 single  0 0 0
connect 321 322 single  0 0 0
connect 323 324 single  0 0 0
connect 325 326 single  0 0 0
connect 327 328 single  0 0 0
connect 329 330 single  0 0 0
connect 331 332 single  0 0 0
connect 333 334 single  0 0 0
connect 335 336 single  0 0 0
connect 337 338 single  0 0 0
connect 339 340 single  0 0 0
connect 341 342 single  0 0 0
connect 343 344 single  0 0 0
connect 345 346 single  0 0 0
connect 347 348 single  0 0 0
connect 349 350 single  0 0 0
connect 351 352 single  0 0 0
connect 353 354 single  0 0 0
connect 355 356 single  0 0 0
connect 357 358 single  0 0 0
connect 359 360 single  0 0 0
connect 361 362 single  0 0 0
connect 363 364 single  0 0 0
connect 365 366 single  0 0 0
connect 367 368 single  0 0 0
connect 369 370 single  0 0 0
connect 371 372 single  0 0 0
connect 373 374 single  0 0 0
connect 375 376 single  0 0 0
connect 377 378 single  0 0 0
connect 379 380 single  0 0 0
connect 381 382 single  0 0 0
connect 383 384 single  0 0 0
```

```
connect 385 386 single  0 0 0
connect 387 388 single  0 0 0
connect 389 390 single  0 0 0
connect 391 392 single  0 0 0
connect 393 394 single  0 0 0
connect 395 396 single  0 0 0
connect 397 398 single  0 0 0
connect 399 400 single  0 0 0
connect 401 402 single  0 0 0
connect 403 404 single  0 0 0
connect 405 406 single  0 0 0
connect 407 408 single  0 0 0
connect 409 410 single  0 0 0
connect 409 411 single  0 0 0
connect 409 412 single  0 0 0
connect 409 413 single  0 0 0
connect 409 414 single  0 0 0
connect 409 415 single  0 0 0
connect 2 341 resonant  0 0 1
connect 16 343 resonant  0 0 1
connect 18 381 resonant  0 1 0
connect 22 391 resonant  1 0 0
connect 30 337 resonant  0 0 1
connect 32 385 resonant  1 0 0
connect 44 339 resonant  0 0 1
connect 50 379 resonant  0 1 0
connect 64 403 resonant  1 0 0
connect 74 397 resonant  0 1 0
connect 102 405 resonant  1 0 0
connect 106 395 resonant  0 1 0
connect 116 393 resonant  0 1 0
connect 120 407 resonant  1 0 0
connect 148 399 resonant  0 1 0
connect 158 401 resonant  1 0 0
connect 170 335 resonant  0 0 1
connect 172 377 resonant  0 1 0
connect 184 333 resonant  0 0 1
connect 190 387 resonant  1 0 0
connect 198 331 resonant  0 0 1
connect 200 389 resonant  1 0 0
connect 204 383 resonant  0 1 0
connect 212 329 resonant  0 0 1

library uff

dump MFU-4-cryst-SF6.grs
output movie arc MFU-4-cryst-SF6
```


References:

[1] Blaine, R. L.; Hahn, B. K. *J. Therm. Anal. Calorim* **1997**, *54*, 695-704.
